# Occurrence, Impact, and Multilocus Sequence Analysis of Alder Yellows Phytoplasma Infecting Common Alder and Italian Alder in Southern Italy

**DOI:** 10.3390/microorganisms12061140

**Published:** 2024-06-04

**Authors:** Carmine Marcone, Roberto Pierro, Carmine Palmieri

**Affiliations:** Department of Pharmacy, University of Salerno, I-84084 Fisciano, Italy

**Keywords:** *Alnus* spp., 16SrV group, PCR, yellows diseases, symptomatology, decline, phylogeny, *map* genotypes, *imp* gene

## Abstract

Alder yellows (ALY) phytoplasma (16SrV-C) is associated with ALY, a disease of several *Alnus* (alder) species in Europe and *A. rubra* in North America. In all affected species, the symptoms are similar. However, latent infections are common. ALY phytoplasma includes different strains which may be occasionally transmitted to grapevines leading to some grapevine yellows diseases. In the current study, visual symptom assessment and PCR-based methods using universal and group-specific phytoplasma primers were used to update and extend knowledge on the occurrence, impact, and genetic diversity of ALY phytoplasma in declining and non-symptomatic *A. glutinosa* and *A. cordata* trees in the Basilicata and Campania regions of southern Italy. ALY phytoplasma was detected in 80% of alder trees examined. In symptomatic trees, no other cause of disease was observed. More than half of alder trees that tested phytoplasma-positive proved to be latently infected. A considerable genetic variability was observed among the newly recorded ALY phytoplasma strains in southern Italy in almost of the genes examined. These included 16S rRNA, 16S/23S rDNA spacer region, ribosomal protein *rpsV* (*rpl22*) and *rpsC* (*rps3*), *map*, *imp,* and *groEL* genes. Eleven new genotypes were identified at *map* gene sequence level. However, the genetic differences observed were not related to plant host species, geographical origin, and symptoms shown by infected alder trees. Also, this study indicates that ALY phytoplasma is more widespread than previously thought.

## 1. Introduction

Members of the genus *Alnus* (alder) (family Betulaceae) are fast-growing tree species distributed throughout the northern temperate zone. They are pioneer species, able to colonize vacant lands rapidly and to form mixed forests as other tree species appear in their wake. Alder trees greatly improve soil fertility through their symbiotic relationship with the nitrogen-fixing bacterium *Frankia alni*. Therefore, they can grow in nutrient-poor soils. Within the genus *Alnus*, the common alder (*A. glutinosa*)—also known as black alder, European alder, or European black alder—native to most of Europe, southwest Asia, and northern Africa, and widely introduced elsewhere, has a considerable ecological and economic value. This species is commonly found near streams, rivers, ponds, lakes, and wetlands. It provides food and shelter for wildlife and valuable wood, is used as a pioneer in ecological succession, to stabilize river banks through its root system, to assist in flood control, to purify water in waterlogged soils, to moderate the temperature and nutrient status of water bodies, and as natural firebreaks. The species is also grown as a specimen tree in parks and gardens. The Italian alder (*A. cordata*), native to the southern Apennines (Campania, Basilicata, and Calabria) and north-eastern Corsica Mountains, which has been introduced in several other Italian regions and European countries, is a multipurpose species that thrives on much drier soils than most other alder species, and grows rapidly even under very unfavorable circumstances, which render this species extremely valuable for landscape planting on difficult sites. It is also used as a fast-growing windbreak.

Taxonomically, phytoplasmas are placed in the class *Mollicutes* and are currently classified within the provisional genus ‘*Candidatus* Phytoplasma’ based primarily on 16S rDNA sequence analysis [1]. Within this genus, several distinct groups (or 16Sr groups) and subgroups have been established by restriction fragment length polymorphism (RFLP) analysis of PCR-amplified rDNA sequences [2,3]. Each 16Sr group is considered to represent at least one distinct species under the provisional taxonomic status ‘*Candidatus*’. Also, multilocus sequence analysis of less-conserved, housekeeping genes is widely used for identification of genetically close but pathologically and/or epidemiologically distinct strains within a given phytoplasma taxon [2,3]. Multilocus sequence analysis of some housekeeping genes is recommended for description of a new ‘*Candidatus* Phytoplasma’ species when the sole 16S rDNA sequence does not support species differentiation [2,3].

Alder yellows (ALY) is a phytoplasma disease of *Alnus* species which is known to occur in several European countries (for reviews see [4,5]). It has also been observed in Washington state (USA) and Ontario (Canada) on red alder (*A. rubra*) [6,7,8,9]. Symptoms of ALY include yellowing, premature autumn coloration, sparse foliage, small leaves, shoot proliferation, stunting, dieback, and decline. Latent infections are also common [6,9,10,11,12,13,14,15,16,17]. ALY is associated with the ALY phytoplasma, a member of the elm yellows (EY) phytoplasma group or 16SrV group, subgroup 16SrV-C [18]. Other members of this group are phytoplasmas causing diseases of trees and shrubs such as EY, flavescence dorée (FD) of grapevine, Palatinate grapevine yellows (PGY), spartium witches’-broom (SpaWB), rubus stunt (RuS), blackberry witches’-broom, cherry lethal yellows, flowering cherry decline, sweet cherry virescence, peach yellows in India, jujube witches’-broom, balanites witches’-broom, Japanese raisin witches’-broom, *Bischofia polycarpa* witches’-broom and *Styphnolobium japonicum* (previously known as *Sophora japonica*) witches’-broom, and phytoplasmas infecting *Clematis vitalba* in Europe and hemp dogbane (*Apocynum cannabinum*) in New York state, respectively [2,18,19,20,21,22,23,24]. Similarly to other members of the EY group, ALY phytoplasma is a homogeneous pathogen at rDNA sequence level. However, a considerable genetic diversity within this pathogen has been observed in less-conserved genes, including *map* [18,25,26,27,28]. On the basis of *map* sequences, ALY phytoplasma strains do not form a homogeneous phylogenetic group but are distributed in various clusters, which comprise FD, PGY, and SpaWB phytoplasma strains [24,25,26]. The ALY phytoplasma is spread among alder trees by the leafhoppers *Oncopsis alni*, *Orientus ishidae*, *Allygus mixtus,* and *Allygus modestus* [29,30,31]. Some ALY phytoplasma strains may be occasionally transmitted through the mentioned leafhoppers from infected alder trees to grapevine leading to grapevine yellows (GY) diseases such as FD and PGY [24,25,32,33,34,35,36].

Most of the foliar and decline symptoms associated with ALY disease are similar to those of phytophthora root and collar rot of alder caused by the chromista *Phytophthora alni* subspecies *alni* and its variants [37], and to those of alder dieback associated either with the mitosporic coelomycete *Phomopsis alnea* or the bacterium *Erwinia alni* [38,39]. However, in addition to the mentioned symptoms, alder trees affected by phytophthora root and collar rot show tongue-shaped necroses of the inner bark and the cambium which extend up to 3 m from the stem base with tarry or rusty spots on the surface of the bark. Dieback-diseased alder trees show elongated cankers along stems, lateral branches and twigs with small, black pustules (pycnidia) protruding through the brown-discolored bark epidermis when infected by *P. alnea,* and water-soaked, dark-brown cankers with a dark-watery liquid oozing from small cracks in the cankers in the case of infections by *E. alni* [37,38,39]. These symptoms and signs are not associated with ALY phytoplasma infections. ALY disease was first reported in southern Italy on common alder and Italian alder in 1994 on the basis of symptomatology, fluorescence, and electron microscope observations and PCR assays. The detected phytoplasma was characterized by RFLPanalysis of PCR-amplified rDNA using *Alu*I restriction endonuclease [11]. Later, a few strains of the ALY phytoplasma recorded in southern Italy were further examined through sequence and extensive RFLP analyses of rDNA and various less-conserved genes [18,25,40,41]. The southern Italian ALY phytoplasma was also transmitted via dodder from diseased alder trees to the experimental host *Catharanthus roseus* (periwinkle), in which it induces symptoms of yellowing, reduced vigor, and small flowers [42].

A major objective of this study was to update and extend knowledge on the occurrence, impact, and genetic diversity of ALY phytoplasma occurring in southern Italy. For this purpose, a survey was carried out in some major alder-growing areas, including those which had previously not been investigated. A special attention was given to visual symptoms assessment in order to make sure that yellowing and decline symptoms were not induced by fungal and/or bacterial infections. Also, multilocus sequence analysis employing genes with varying degrees of genetic variability was used.

## 2. Materials and Methods

### 2.1. Plant Samples, Symptom Evaluation, and Phytoplasma Reference Strains

**Figure 1:**
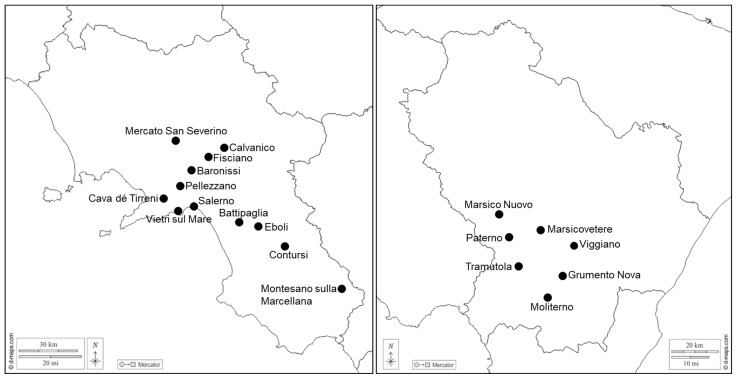
Locations of sampling in the Campania (**left**) and Basilicata (**right**) regions of southern Italy. Maps were generated with d-maps.com https://d-maps.com/pays.php?num_pay=397&num_pag=1&lang=en (accessed on 12 May 2024). Individual maps were assembled and labeled with the software program Photoshop CS3 (www.adobe.com (accessed on 12 May 2024)).

Sampling of symptomatic and non-symptomatic common alder and Italian alder trees was performed in several locations of the Basilicata and Campania regions of southern Italy from 2019 through 2023, mainly during summer and fall. Trees were repeatedly inspected for the presence of ALY symptoms and for symptoms and signs of phytophthora root and collar rot and alder dieback associated either with fungal or bacterial infections. A total of 255 alder trees were sampled from their natural habitats: 150 trees were sampled in the Agri valley of the Basilicata region near Marsico Nuovo (15 trees), Paterno (22 trees), Marsicovetere (23 trees), Tramutola (28 trees), Viggiano (18 trees), Grumento Nova (30 trees), and Moliterno (14 trees), whereas 105 trees were sampled in the Campania region near Montesano sulla Marcellana (15 trees), Contursi (20 trees), Eboli (10 trees), Battipaglia (8 trees), Salerno (9 trees), Vietri sul Mare (12 trees), Cava dé Tirreni (5 trees), Pellezzano (3 trees), Baronissi (8 trees), Mercato San Severino (5 trees), Fisciano (6 trees), and Calvanico (4 trees) (Figure 1). Many of these trees were sampled repeatedly for re-examination. Of the trees sampled, two-thirds consisted of common alder, while the remaining were Italian alder. In addition to these field-collected samples, DNA samples from the following periwinkle-maintained phytoplasma reference strains were included in this work for comparison: ULW, elm yellows from France collected by G. Morvan, Avignon-Montfavet, France; EY1, elm yellows from North America collected by W.A. Sinclair, Ithaca, NY, USA; and RUS, rubus stunt and ALY, alder yellows, both from southern Italy, collected by C. Marcone, Fisciano (Salerno), Italy. More information on these strains and their taxonomic positions is given elsewhere [41,42,43]. DNA samples extracted from SpaWB-affected Spanish broom plants, grown in southern Italy and infected by 16SrV-C subgroup phytoplasma [40,44], as well as from FD-affected grapes, grown in northern Italian vineyards, infected by 16SrV-D subgroup and 16SrV-C subgroup phytoplasmas (provided by F. Quaglino, University of Milan, Milan, Italy), were also used for comparisons. One-to-two-year-old seedlings of common alder that were grown in an insect-proof screenhouse were used as healthy controls.

### 2.2. DNA Isolation and PCR Amplification

For DNA extraction, petioles, midribs, and/or phloem preparations from stem portions or roots approximately 5 cm in diameter, collected from symptomatic and non-symptomatic randomly selected alder trees, were used. Phloem tissue was prepared as described [45]. DNA was isolated from approximately 1.0 g of fresh tissue using a phytoplasma enrichment procedure as described previously [46].

Phytoplasmal rDNA was amplified by PCR using both universal and group-specific primers. The universal phytoplasma primer pair P1/P7 [47] amplified a fragment of approximately 1800 bp in length that extends from the 5′ end of the 16S rRNA gene, through the intergenic region (16S/23S rDNA spacer region), to the 5′ region of the 23S rDNA. Another universal primer pair, P1A/P7A, amplified a fragment of almost the same length encompassing the same genes [18]. Primer pair fB1/rULWS, which primes in the 5′ region of the 16S rRNA gene and in the 16S/23S rDNA spacer region, specifically amplified a fragment of about 1650 bp from the EY-group phytoplasmas [48], whereas primer pair R16(V)F1/R1 [49] specifically amplified a 1100 bp 16S rDNA fragment from the EY-group phytoplasmas. In nested PCR assays, initial amplification (‘first round’) was carried out with primer pair P1/P7. The products obtained were reamplified either with the universal primers P1A/P7A or with group-specific primers fB1/rULWS and R16(V)F1/R1. Ribosomal protein (rp) gene operon was amplified using the primer pair rp(V)F1/rpR1 specific to the EY-group phytoplasmas followed by another EY group-specific primer pair, rp(V)F1A/rp(V)R1A [18]. The nested PCR with primer pair rp(V)F1A/rp(V)R1A yielded a 1200 bp DNA fragment covering the region with rp genes *rpsV* (*rpl22*) and *rpsC* (*rps3*). Non-ribosomal secY-map locus (*map* gene), which encodes a methionine aminopeptidase, was amplified using the primer pair FD9f5/MAPr1 followed by the primer pair FD9f6/MAPr2 [25]. Both primer pairs are specific for the EY-group phytoplasmas. *Imp* gene, which encodes an immunodominant surface protein, and *groEL* gene encoding the heat-shock protein GroEL, were amplified through the EY group-specific primer pairs fEY_imp/rpyrG and fEY_groEL/rEY_groEL, respectively [50]. Primer pair fEY_imp/rpyrG yielded a 675 bp DNA fragment, whereas primer pair fEY_groEL/rEY_groEL amplified a fragment of about 880 bp in length. Details of primers used in this study are given in Table S1.

PCR amplifications were performed in a 50 µL reaction containing 125 µM of the four dNTPs, 0.5 µM of each primer, 1 U of Dream Taq DNA polymerase, 1× polymerase buffer (both Thermo Fisher Scientific, Waltham, MA, USA), and 5 µL of template DNA (100–200 ng), and water. The mixture was subjected to 35 cycles in a thermocycler Primus 25 (Peqlab-Life Science, Erlangen, Germany) at the following incubations: 30 s denaturation at 95 °C, 60 s annealing at 55 °C (50 °C with primer pairs fB1/rULWS, R16(V)F1/R1, rp(V)F1/rpR1, and rp(V)F1A/rp(V)R1A, 52 °C with primer pairs FD9f5/MAPr1 and FD9f6/MAPr2, 54 °C with primer pair fEY_imp/rpyrG, 59 °C with primer pair fEY_groEL/rEY_groEL), and 90 s extension (60 s with the primer pairs R16(V)F1/R1, rp(V)F1/rpR1, rp(V)F1A/rp(V)R1A, FD9f5/MAPr1, FD9f6/MAPr2, fEY_imp/rpyrG, and fEY_groEL/rEY_groEL) at 72 °C (66 °C with primer pairs FD9f5/MAPr1 and FD9f6/MAPr2). The final step was 10 min at 72 °C. For amplification with nested primers, either 1 μL of undiluted PCR products or 3 μL of 1:40 dilution obtained in the initial amplification was used as a template in the reaction mixture described above. Five µL of PCR products was analyzed by electrophoresis in a 1.5% horizontal agarose gel in TAE buffer (40 mM Tris-acetate, 1 mM EDTA, pH 8.0) in the presence of 0.5 µg/mL ethidium bromide. DNA bands were visualized using a UV transilluminator.

### 2.3. Actual and Virtual RFLP Analyses

Ten microliters of PCR products of a representative number of positive samples from each location obtained with either the primer pair P1/P7 or primer pairs P1A/P7A and fB1/rULWS were separately digested with *Taq*I and *Bfa*I restriction endonucleases following the manufacturer’s instructions (Thermo Fisher Scientific). Seven to twelve microliters of the digests were used to resolve the restriction fragments on vertical 5% polyacrylamide gel in TBE buffer (45 mM Tris-borate, 1 mM EDTA, pH 8). After electrophoresis, the DNA was visualized with ethidium bromide as described above. Molecular weights were determined using the 1 Kb Plus DNA ladder (Thermo Fisher Scientific). Sequences of P1A/P7A and rp(V)F1A/rp(V)R1A amplicons obtained from ALY-infected alder trees (see Section 2.4) were subjected to in silico (=virtual) RFLP analysis through the pDRAW32 program developed by AcaClone Software, version 1.1.147 (http://www.acaclone.com (accessed on 12 May 2024)) using key restriction enzymes for differentiation of 16SrV group phytoplasmas [18,51,52]. The resulting RFLP patterns were compared to those of phytoplasma reference strains of the 16SrV group whose sequences are available in GenBank. Also, virtual RFLP analysis using 17 restriction enzymes of the 16S rDNA sequences corresponding to the R16F2n/R2 fragments of ALY phytoplasma strains recorded in the present study was performed through the *i*PhyClassifier online tool [53] at http://plantpathology.ba.ars.usda.gov/cgi-bin/resource/iphyclassifier.cgi (accessed on 12 May 2024).

### 2.4. DNA Sequencing and Phylogenetic Analysis

For sequencing, the P1A/P7A, rp(V)F1A/rp(V)R1A, FD9f6/MAPr2, fEY_imp/rpyrG, and fEY_groEL/rEY_groEL PCR products were purified using the PureLink PCR Purification Kit (Invitrogen, Waltham, MA, USA) and sequenced directly. Sequencing of both strands was performed by a commercial service (BMR Genomics, University of Padua, Padua, Italy). Primers for sequencing PCR products were the same as for PCR amplification. The obtained sequences were assembled and edited using DNASTAR’s LaserGene software (DNASTAR), version 7.1 and consensus sequences were generated. These were then used as query sequences in a BLAST search [54] carried out online at https://blast.ncbi.nlm.nih.gov/Blast.cgi (accessed on 12 May 2024). Sequence alignments were performed by using CLUSTAL W, using DNASTAR’s LaserGene software (DNASTAR). The GenBank accession nos. of sequences determined in this study are as follows: PP097677 to PP097690 for rDNA, PP125318 to PP125344 for *rpsV* (*rpl22*) and *rpsC* (*rps3*), PP135471 to PP135498 for *map*, PP230419 to PP230443 for *Imp*, and PP230394 to PP230418 for *groEL* sequences. Other sequences retrieved from the GenBank database and used in this study are given in Tables S2 and S4–S7. Phylogenetic and molecular evolutionary analyses were conducted using the neighbor-joining program of the genetic analysis software MEGA, version 11 [55]. The data were resampled 1000 times and the bootstrap percentage values were given at the nodes of the tree. Neighbor-joining and maximum likelihood methods yielded similar results in tree construction. Phylogenetic distances were calculated by pairwise comparison.

## 3. Results

### 3.1. Incidence and Impact of ALY Phytoplasma Infections

**Figure 2:**
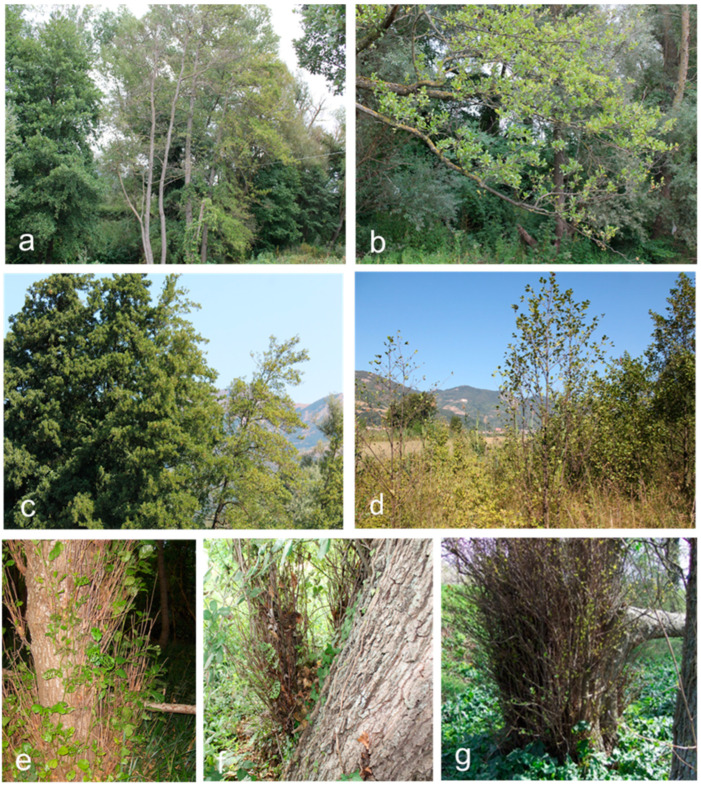
Diseased *Alnus glutinosa* (common alder) trees associated with alder yellows phytoplasma infections. (**a**) Trees showing foliar yellowing and decline (center). Healthy-looking trees are on both sides. (**b**) Branches with severe yellowing, small leaves, and sparse foliage. (**c**) Trees showing yellowing, decline, and stunted branches. Healthy-looking tree on the left. (**d**) Young trees with sparse foliage, premature autumn coloration, premature defoliation, and slender shoots. (**e**–**g**) Shoot proliferation at the base of trunk of several-year-old trees.

With universal primer pairs P1/P7 and P1A/P7A and group-specific primer pairs fB1/rULWS and R16(V)F1/R1, the target DNA was amplified from 204 out of 255 alder trees examined. Of these, all symptomatic trees (99 trees) and 105 non-symptomatic trees tested phytoplasma-positive. As expected from previous work [56,57,58], nested PCR was more sensitive than direct (‘one-round’) PCR. Symptomatic trees scored phytoplasma-positive with both direct and nested PCR assays. However, in samples from non-symptomatic trees, visible PCR products could be observed only when amplification products obtained with primers P1/P7 were re-amplified with either universal primer pair P1A/P7A or group-specific primer pairs fB1/rULWS and R16(V)F1/R1. Similarly, primer pairs rp(V)F1/rpR1 and rp(V)F1A/rp(V)R1A, FD9f5/MAPr1 and FD9f6/MAPr2, and fEY_imp/rpyrG and fEY_groEL/rEY_groEL showed the same amplification spectrum as primer pairs directed to rDNA sequences. All phytoplasma reference strains, including SpaWB and FD phytoplasmas, yielded an amplification product of the expected size with any of the primer pairs employed. Neither by direct nor by nested PCR assays was DNA amplified from template DNA isolated from healthy control plants. The most common symptoms observed on diseased alder trees were light-green leaves, yellowing, smaller leaves than normal, reduced vigor, sparse foliage, stunting, and decline. In some cases, foliar symptoms were limited to a single branch or part of it. Then, they gradually spread to other parts of the tree. In addition, abnormal branching in the tree canopy, premature autumn coloration, growth of premature shoots conferring a witches’-broom appearance, and shoot proliferation at the base of trunks were recorded in some instances (Figure 2 and Figure S1). None of the alder trees examined showed typical symptoms and signs of *P. alni*, *P. alnea,* and *E. alni* infections.

### 3.2. Actual and Virtual RFLP Analyses

**Figure 3:**
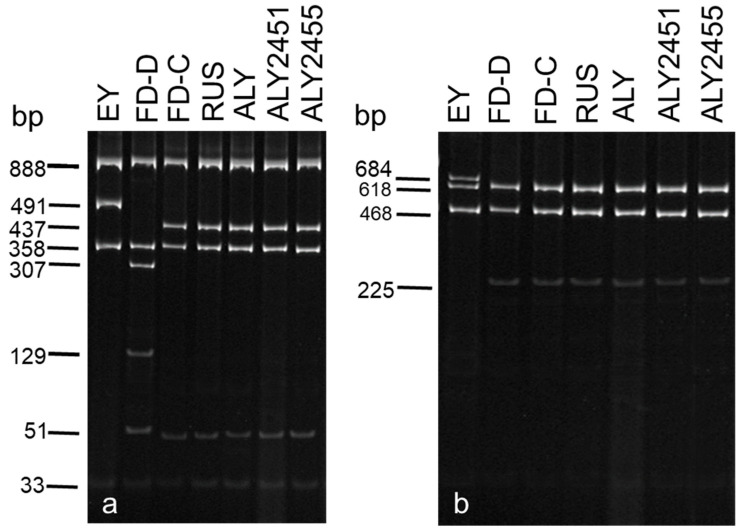
(**a**) *Taq*I and (**b**) *Bfa*I actual restriction profiles of rDNA P1A/P7A fragments from alder yellows (ALY) phytoplasma strains and other 16SrV group phytoplasmas. EY, elm yellows (strain EY1); FD-D, flavescence dorée (subgroup 16SrV-D); FD-C, flavescence dorée (subgroup 16SrV-C); RUS, rubus stunt; ALY, alder yellows; ALY2451 and ALY2455, newly recorded ALY phytoplasma strains in southern Italy. The gel was photographed with the ImageQuant LAS 4000 system, version 1.2 (www.gelifesciences.com (accessed on 12 May 2024)) and cropped with Photoshop CS3 (www.adobe.com (accessed on 12 May 2024)).

Following digestion of P1/P7 and/or P1A/P7A PCR products with *Taq*I restriction endonuclease, all phytoplasma-positive samples from alders showed the same restriction profile as the periwinkle-maintained strain ALY of the ALY phytoplasma (Figure 3a). This profile was identical to those of strains SpaWB and RUS and resulted from the presence of four cleavage sites in the amplified sequences. Strains ULW and EY1 and FD showed different restriction profiles which resulted from the presence of three and five cleavage sites, respectively. After digestion of fB1/rULWS PCR products with *Taq*I restriction enzyme, all phytoplasma-positive samples from alders showed the same restriction profile as ALY, SpaWB, ULW, EY1, and RUS reference strains. This similarity is due to the fact that the *Taq*I restriction site in the 16S/23S rDNA spacer region that enables differentiation of the 16SrV-C subgroup phytoplasmas from the EY agent [40], was not present in the rDNA fragment amplified with primer pair fB1/rULWS. However, FD strain showed differences to the other phytoplasmas examined which were similar to those obtained following digestion of P1/P7 and/or P1A/P7A amplicons. When P1/P7 and/or P1A/P7A and fB1/rULWS PCR products were digested with *Bfa*I restriction endonuclease, all phytoplasma-positive samples from alders showed the same restriction profile as phytoplasma reference strains ALY, RUS, SpaWB, and FD reference strains. This profile, which resulted from the presence of three restriction sites, differed from that of ULW and EY1 reference strains, which are characterized by the presence of two restriction sites (Figure 3b).

Virtual RFLP analysis of P1A/P7A sequences obtained from the newly recorded ALY phytoplasma strains in southern Italy, hereafter collectively referred to as ‘NR-SI-ALY strains’, (see Section 3.3) with *Taq*I and *Bfa*I restriction endonucleases showed uniform restriction profiles with each endonuclease. The *Taq*I profile was identical to those of strains ALY-SI, FD-C(FD70), and RUS but different from those of strains EY1 and FD-D(FD1487), respectively (Figure S2a). The *Bfa*I profile was identical to those of strains ALY-SI, FD-C(FD70), FD-D(FD1487), and RUS but different from that of strain EY1 (Figure S2b). Expected sizes based on virtual restriction analysis were in agreement with fragment sizes obtained by enzymatic RFLP analysis of P1A-P7A amplicons (Figure 3 and Figure S2).

Virtual RFLP analysis of the 16S rDNA sequences encompassing the entire F2nR2 fragment showed that the NR-SI-ALY strains are members of the 16SrV-C subgroup. Of these, strains ALY2451, ALY2455, ALY2457, ALY2460, ALY2461, ALY2462, ALY2464, ALY2555, ALY2556, ALY2614, ALY2917, ALY2919, and ALY2924 had RFLP profiles identical to those of the reference strain ALY882, showing a pattern similarity coefficient of 1.00, whereas strain ALY2518 had RFLP profiles which were most similar to those of the reference strain ALY882, with a pattern similarity coefficient of 0.98 (Figure S3). Therefore, phytoplasma strain ALY2518 is a variant of the 16SrV-C subgroup.

Virtual RFLP analysis of rp(V)F1A/rp(V)R1A sequences with *Hpa*II and *Dra*I resulted in the presence of the same RFLP profiles from 26 out of 27 NR-SI-ALY strains examined with each enzyme. These profiles were identical to those of strains ALY, ALY882, and ALY1068 and resulted from the presence of one and seven restriction sites, respectively (Table S3). Strain ALY2465 did not show any *Hpa*II restriction sites, giving the same profile as strain FD-(FD-C). Following virtual digestion with *Taq*I, strains ALY2413, ALY2436, ALY2450, ALY2454, ALY2456, ALY2459, ALY2465, ALY2486, ALY2515, ALY2518, ALY2555, ALY2587, ALY2588, ALY2611, ALY2917, ALY2918, ALY2921, ALY2923, ALY2924, ALY3016, and ALY3041 showed the same restriction profile as strains ALY, ALY882, ALY1068, and FD-(FD-C). This profile was characterized by the presence of one restriction site. Strains ALY2438, ALY2460, ALY2463, ALY2606, ALY2610, and ALY3040 showed the same *Taq*I restriction profile as strains FD-(FD-D) and ALY-(AldWB), which had two restriction sites. After virtual digestion with *Alu*I, all NR-SI-ALY strains examined showed the same *Alu*I restriction profile as strains ALY, ALY1068, FD-(FD-C), SpaWB-(SpaWB229), and ALY-(AldWB). This profile was due to the presence of 10 restriction sites. All NR-SI-ALY strains except strain ALY2465 showed the same *Mse*I restriction profile as strains ALY, ALY1068, and SpaWB-(SpaWB229), which resulted from the presence of 27 restriction sites. Strain ALY2465 had a distinct *Mse*I restriction profile due to the presence of an additional site at position 840. When rp(V)F1A/rp(V)R1A fragments were virtual digested with *Ssp*I, 13 strains (ALY2413, ALY2450, ALY2454, ALY2459, ALY2486, ALY2515, ALY2518, ALY2555, ALY2917, ALY2918, ALY2923, ALY2924, and ALY3041) showed a restriction profile identical to that of strains ALY and ALY1068. This profile resulted from the presence of five restriction sites. The remaining strains (ALY2436, ALY2438, ALY2456, ALY2460, ALY2463, ALY2465, ALY2587, ALY2588, ALY2606, ALY2610, ALY2611, ALY2921, ALY3016, and ALY3040) had restriction profiles identical to that of strain ALY882 which resulted from the presence of four restriction sites. When the same rp fragments were virtual digested with *Tsp509*I, strains ALY2413, ALY2459, ALY2460, ALY2486, ALY2518, ALY2555, ALY2587, ALY2611, ALY2917, ALY2918, ALY2921, ALY2924, and ALY3041 showed the same restriction profile as strains ALY and ALY1068. This profile resulted from the presence of 18 restriction sites and differed from that shown by strains ALY2436, ALY2438, ALY2450, ALY2454, ALY2456, ALY2463, ALY2465, ALY2515, ALY2588, ALY2606, ALY2610, ALY2923, ALY3016, and ALY3040 which was characterized by the presence of 17 restriction sites and was identical to that of strain ALY882. None of the rp(V)F1A/rp(V)R1A sequences had *Hae*III and *Hha*I restriction sites.

### 3.3. DNA Sequencing and Phylogenetic Analysis

Nucleotide sequence analysis of PCR-amplified rDNA revealed that the NR-SI-ALY strains shared a 16S rDNA sequence similarity which varied from 97.6% (ALY2461 versus ALY2555) to 99.8% (ALY2460 versus ALY2919 and ALY2464 versus ALY2919) (Table S4). Sequence comparisons of the mentioned strains with ALY strains which were previously recorded in alder, either in southern Italy or other European countries, whose full-length or nearly full-length 16S rDNA sequences are available from the GenBank database, showed sequence similarity values between 98.3% and 99.9%. Strains ALY2451, ALY2461, and ALY2556 showed a 100% 16S rDNA sequence similarity with a strain detected in alder in Lithuania [12]. However, for the latter strain, the 16S rDNA sequence available from the GenBank database is only 1203 bp in length. Comparisons with other 16SrV-C subgroup members showed 16S rDNA sequence similarity values ranging from 98% (ALY2555 versus FD-[FD-D]) to 99.9% (ALY2451 and ALY2917 versus MugWB-[MugWB], FD-[FD-C] and FD-[FD70]). In interspecific comparisons of NR-SI-ALY strains with other 16SrV subgroup phytoplasmas, namely ‘*Candidatus* (*Ca*.) Phytoplasma ulmi’, ‘*Ca*. Phytoplasma ziziphi, ‘*Ca*. Phytoplasma rubi’, and ‘*Ca*. Phytoplasma balanitae’, differences in 16S rDNA sequences were 0.3–1.9%, 1.0–2.0%, 0.6–2.2%, and 2.0–3.5%, respectively. Phytoplasmas from other 16Sr groups differ from NR-SI-ALY strains in more than 4.5% of 16S rDNA positions. The phylogenetic relationships of NR-SI-ALY strains based on 16S rDNA sequences to each other and to other phytoplasmas are depicted in Figure S4. NR-SI-ALY strains clustered tightly together along with other strains infecting alder and other 16SrV-C subgroup phytoplasmas. At 16S/23S rDNA spacer region sequence level, NR-SI-ALY strains had similarity values between 89.4% and 100% (Table S5). Strains ALY2451, ALY2460, and ALY2462 proved to be identical to ALY-(AldWB), MugWB-(MugWB), FD-(FD70), FD-(34c2), FD-(CH), and FD-(FD1487) phytoplasmas. In phylogenetic analysis, some NR-SI-ALY strains clustered together, whereas the remaining clustered with other 16SrV-C subgroup phytoplasmas (Figure S5).

Sequence analysis of rp genes *rpsV* (*rpl22*) and *rpsC* (*rps3*) of 27 NR-SI-ALY strains, revealed similarity values ranging from 99% to 100% (Table S6). These strains shared 98.5–99.9% and 98.2–99.6% rp sequence similarity with other ALY strains and other 16SrV-C subgroup members (FD-D, FD-C, SpaWB229, SpaWB251, and HD1), respectively. Sequence similarity to other 16SrV subgroup members was lower, being 98.1–98.9%, 97.1–98.2% and 95.8–96.5% for 16SrV-F (RUS, RuS400, RuS971, and RuSR19), 16SrV-A (EY1, EY125, EY626, EY627, EY-NK16, and EY24-SRB), and 16SrV-B (CLY5, PY-In, and JWB) subgroup phytoplasmas, respectively. Phylogenetic relatedness of NR-SI-ALY strains at rp gene sequence level to each other and to other 16SrV subgroup phytoplasmas are shown in Figure S6.

Sequence analysis of *map* gene amplicons showed similarity values which ranged from 98.5% to 100% among the NR-SI-ALY strains examined (Table S7). These strains shared 97.3–100%, 97.9–100% and 97.5–99.4% *map* gene sequence similarity with other ALY strains, FD, and PGY strains, respectively. Also, they shared 97.9–98.8% and 96.9–97.5% sequence similarity with other 16SrV-C subgroup members such as SpaWB and HD phytoplasmas, respectively. More distantly related were ‘*Ca*. Phytoplasma rubi’ and ‘*Ca*. Phytoplasma ulmi’ which shared 97.6–98.2% and 96.6–97.3% *map* gene sequence similarity with NR-SI-ALY strains, respectively. Within the NR-SI-ALY strains, ALY2514, ALY2919, and ALY2921 proved to be identical and showed a 100% sequence similarity with strain AI-AL4, previously detected in *A. glutinosa* in Tuscany, central Italy, which corresponds to the *map* genotype M113 [24] (Table S8). Strain ALY3043 was undistinguishable from the periwinkle-maintained ALY strain and from the FD strain VF-09-112 previously identified in *Vitis vinifera* in France, both corresponding to the *map* genotype M36 [24,25]. Strains ALY2515, ALY2587, ALY2599, ALY2600, ALY2603, ALY2689, ALY2735, ALY2924, ALY3014, ALY3015, ALY3018, ALY3021, and ALY3040 were identical, showing sequence similarity values of 100%. All these strains differed by a single nucleotide polymorphism (SNP) (C instead of A) at position 593 from the ALY strain AI-04-3-13 previously detected in southern Italy corresponding to the *map* genotype M35 [24,25] (Table S9). Strain ALY2590 differed by an SNP (A instead of G) at position 404 from the ALY strain AI-AL4 (*map* genotype M113) and from strains ALY2514, ALY2919, and ALY2921, whereas strain ALY2604 differed by an SNP (A instead of G) at position 166 from the ALY strain 74-08-MNE previously identified in *A. glutinosa* in Montenegro, corresponding to the *map* genotype M142 [14,28], and by an SNP (C instead of A) at position 604 from the FD-D1448 strain previously identified in hazelnut in Slovenia, corresponding to the *map* genotype M159 [59]. Strain ALY2610 differed by two SNPs (T instead of G and C instead of A, at positions 372 and 593, respectively) from the ALY strain AI-04-3-13 (*map* genotype M35). Strain ALY2687 differed by three SNPs (T instead of G, T instead of C, and C instead of A at positions 216, 313 and 456, respectively) from periwinkle-maintained ALY strain and FD strain VF-09-112 (*map* genotype M36), whereas strain ALY2711 differed by two SNPs (A instead of G and C instead of A at positions 11 and 593, respectively) from the ALY strain AI-04-3-13 (*map* genotype M35). Strains ALY2712, ALY2745, ALY2912, and ALY3016 each differed from the ALY strain AI-04-3-13 (*map* genotype M35) by the same SNP (C instead of A) at position 593 and by an additional distinctly different SNP. Strains ALY2917 and ALY3041 had identical sequences which differed by an SNP (A instead of G) at position 166 from that of ALY strain AI-AL4 (*map* genotype M113). The phylogenetic relationships of NR-SI-ALY strains based on *map* gene sequences to each other and other ALY strains and 16SrV group phytoplasmas are shown in Figure S7. NR-SI-ALY strains clustered tightly together with some previously identified ALY strains.

**Figure 4:**
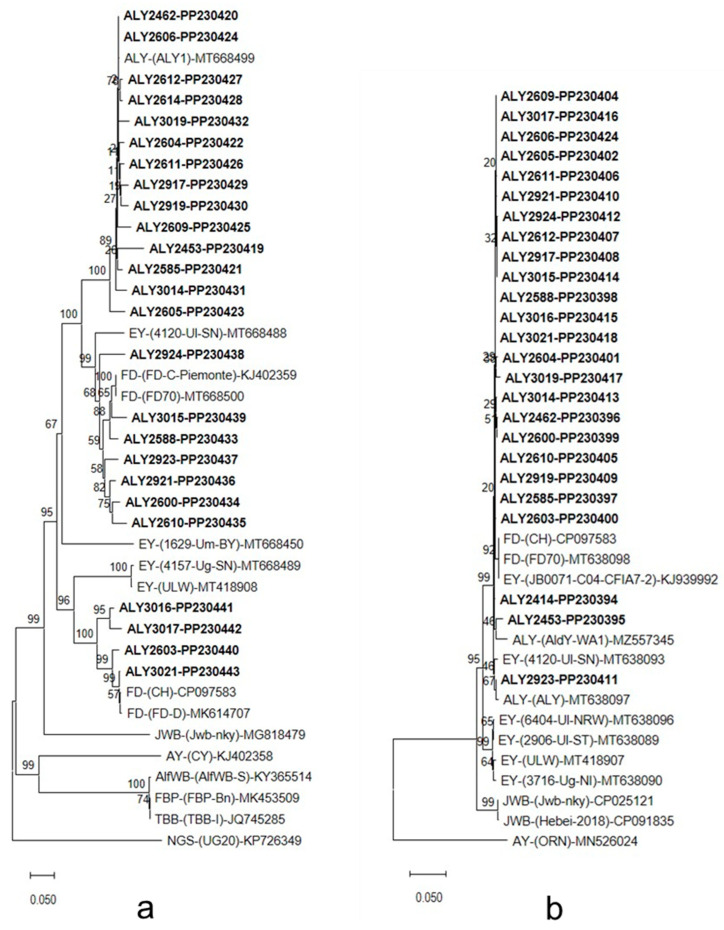
Phylogenetic tree constructed using the neighbor-joining method software MEGA, version XI (www.megasoftware.net (accessed on 12 May 2024)) [55] with *imp* (**a**) and *groEL* (**b**) gene sequences from newly detected ALY phytoplasma strains from southern Italy (in bold type), ALY strains previously detected in alder, and a number of reference phytoplasmas. Napier grass stunt (NGS) phytoplasma strain UG20 (**a**) and aster yellows phytoplasma strain ORN (**b**) were used as the outgroups. Bar represents a phylogenetic distance of 0.05 nucleotide substitutions for site. GenBank accession number is given for each phytoplasma. Bootstrap values are shown on branches of the phylogenetic trees.

Sequence analysis of *imp* gene of 25 NR-SI-ALY strains showed similarity values which varied from 70.2% (ALY2453 versus ALY2603) to 100% (ALY2462 versus ALY2606) (Table S10). The most closely related to NR-SI-ALY strains was the strain ALY1, previously recorded in *A. glutinosa* in Germany [50], which shared 72.1–99.8% *imp* gene sequence similarity with them. NR-SI-ALY strains shared 71–99.6% *imp* gene sequence similarity with FD strains (CH, FD-D, FD-C-Piemonte, and FD70) and were more distantly related to EY strains, sharing with them 67.3–90.8% *imp* gene sequence similarity. In phylogenetic analysis based on *imp* gene sequences, NR-SI-ALY strains formed a monophyletic group which split in two branches, one comprising FD strains FD-(CH) and FD-D (16SrV-D subgroup), the other comprising FD strains FD-C-Piemonte and FD70 (subgroup 16SrV-C) and the German ALY strain ALY1. Both branches diverged in sub-branches, each of which contained strains of the same 16SrV subgroup. Within sub-branches, distinct lineages were resolved as supported by high bootstrap values. Flanked to the clusters comprising NR-SI-ALY strains were EY strains 1629-Um-BY, 4157-Ug-SN, ULW, and 4120-Ul-SN (Figure 4a).

At *groEL* gene sequence level, NR-SI-ALY strains showed similarity values ranging from 98.2% (ALY2453 versus ALY2462) to 100% (ALY2588 versus ALY3016) (Table S11). NR-SI-ALY strains shared 97.7–99.1% and 97.3–98% *groEL* gene sequence similarity with the periwinkle-maintained ALY strain [50] and the strain AldY-WA1, respectively. Other 16SrV group members which were most closely related to NR-SI-ALY strains, were FD-(CH) and FD-(FD70), and EY-(JB0071-C04-CFIA7-2) and EY-(4120-Ul-SN), sharing with them 98–99.5% and 98.3–99.3% *groEL* gene sequence similarity, respectively. Strains EY-(6404-Ul-NRW), EY-(ULW), EY-(2906-Ul-ST), and EY-(3716-Ug-NI) shared 95.4–97.1% *groEL* gene sequence similarity with NR-SI-ALY strains. Phylogeny inferred by *groEL* gene sequences showed a monophyletic origin of NR-SI-ALY strains. They clustered together with the periwinkle-maintained ALY strain, the ALY strain AldY-WA1, FD strains FD-(CH) and FD70, and EY strains 4120-Ul-SN and JB0071-C04-CFIA7-2. Flanked to the clusters of ALY strains were members of the 16SrV-A and -B subgroups (Figure 4b).

## 4. Discussion

In this work, visual symptom assessment and PCR assays using universal and group-specific phytoplasma primer pairs directed to rDNA sequences were employed to detect phytoplasma infections in declining and non-symptomatic *A. glutinosa* and *A. cordata* trees in the Basilicata and Campania regions of southern Italy. On the basis of primer specificity and RFLP and sequence analyses of PCR-amplified rDNA sequences, the detected phytoplasma was assigned to the ALY agent, a member of the 16SrV-C subgroup. Also, multilocus sequence analysis employing genes with varying degrees of genetic variability was used to elucidate the genetic diversity of the ALY phytoplasma occurring in southern Italy. ALY phytoplasma was detected in 80% of alder trees examined. More than half of alder trees that tested phytoplasma-positive proved to be latently infected. The high detection rate recorded in this work is in agreement with data from other investigations in which detection rates ranging from 70% to 100% of ALY phytoplasma infections in *A. glutinosa* were recorded by PCR assays in various European countries [16,24,26,29,30,31,60,61,62]. The widespread occurrence of ALY phytoplasma in southern Italy implies the presence of an abundant and efficient insect vector which easily acquires the pathogen from the highly colonized alder trees and transmits it to other alder trees. Knowledge on these epidemiological aspects is needed for effective disease management. Similarly to previous work, a high percentage of infected alder trees in southern Italy did not show symptoms [6,24,26,29]. Because symptoms and signs typical of fungal and/or bacterial infections were not observed in the present study in symptomatic alder trees that tested phytoplasma-positive, it can be concluded that foliar yellowing, stunting, and decline symptoms exhibited by these trees were incited by the ALY phytoplasma. Most of the observed ALY symptoms were similar or identical to those previously described in southern Italy and other geographic areas from Europe [4,5,6,7,8,10,11,12,13]. The reasons why in nature a high percentage of alder trees infected by the ALY phytoplasma do not develop obvious symptoms were investigated by Berges and Seemüller [63] through graft-inoculation experiments. This study showed that ALY phytoplasma is pathogenic to alder and may induce severe symptoms, but avirulent strains occurring within this taxon account for the latent infections that are widespread in Europe [63]. It is also possible that avirulent strains mediate cross-protection and suppress severe strains since infected alder trees remain symptomless over time in spite of the presence of abundant and efficient insect vectors. Differences in strain virulence and antagonistic interactions among different strains of the same pathogen have been observed for several other phytoplasmas including those infecting trees and shrubs [64,65,66]. It is also possible that the symbiotic *Frankia alni* may compensate the detrimental effects of phytoplasma infections in alder [17]. However, it is unknown whether this bacterium is able to colonize systemically the phloem tissue of alder trees.

A considerable genetic variability was observed among the newly recorded ALY phytoplasma strains in southern Italy (=NR-SI-ALY strains) in almost all of the genes examined. The degrees of genetic variability observed in the 16S rRNA gene, with dissimilarity values of 0.2–2.4%, had previously not been known to occur within the ALY phytoplasma. This finding may imply the occurrence of distinct taxonomic entities within the ALY agent. Similarly to previous findings, some NR-SI-ALY strains proved more closely related to some FD phytoplasma strains than to other alder-infecting phytoplasma strains [25,31]. Also, virtual RFLP analysis of the 16S rDNA sequences enabled the identification of a variant of the 16SrV-C subgroup among the NR-SI-ALY strains. This is the first evidence of a 16SrV-C subgroup variant within the ALY phytoplasma. On the basis of collective profiles obtained through virtual RFLP analysis of rp gene sequences using suitable restriction enzymes, two previously proposed pattern types or subgroups (rpV-H and -D) [18] were recognized among the phytoplasma strains under study. In addition, six new pattern types with distinct RFLP profiles were identified and designated rpV-N, -O, -P, -Q, -R, and -S. Of these, rpV-P is represented by the strain ALY-(AldWB) from Poland whose sequence was available in the GenBank database [27]. At *map* gene sequence level, two previously reported genotypes, namely *map* genotype M36 and *map* genotype M113 [24,25] were recognized among the NR-SI-ALY strains. In addition, eleven new *map* genotypes were identified in the material examined and designated *map* genotype M163 through *map* genotype M173. These new genotypes enabled us to update and extend the previously defined *map* genotype classification of 16SrV-C and -D phytoplasmas occurring in plants and insect vectors [24,25,28,36,59]. The most prevalent genotype recorded in the present study was *map* genotype M163, which included 13 out of 28 strains examined. This genotype is an SNP variant of *map* genotype M35 previously detected in southern Italy [24,25]. The newly identified *map* genotype M165 is closely related to the *map* genotype M159 previously identified in hazelnut in Slovenia, a member of the Map-FD1 cluster [59]. This cluster consists of strains which, due to their transmissibility by the insect vector *Scaphoideus titanus,* are involved in FD outbreaks [24]. FD disease and *S. titanus* have been reported in the Campania region, while *S. titanus* has also been reported in Basilicata [67,68]. In addition, *map* genotype M36 recognized in the present work is also known to occur in grapevine where it is associated with PGY disease [24]. Therefore, it can be concluded that infected alder trees in southern Italy may also serve as reservoirs for the GY agents. The occurrence of a wide array of different *map* genotypes in alder trees in southern Italy further supports data of previous investigations by which a higher diversity of the mentioned genotypes was found in alder trees and *O*. *alni* than in grapevine and *S*. *titanus*, all sampled in various areas of five European countries [24]. Prior to this work, only one and two ALY strains had been examined at *imp* and *groEL* gene sequence level, respectively [9,50]. On the other hand, sequence and phylogenetic analyses of both genes have been widely used to characterize and differentiate strains of ‘*Ca*. Phytoplasma ulmi’ within the 16SrV group [50]. Also, these genes have been used as additional molecular tools for characterization and differentiation of strains of 16SrII and 16SrX groups (the *imp* gene) and 16SrI and 16SrXIV groups (the *groEL* gene) [69,70,71,72,73,74,75]. NR-SI-ALY strains exhibited a greater genetic variability at *imp* gene sequence level than at *groEL* gene level. These data largely confirm the results of previous work which showed that *imp* and *groEL* genes are two markers of different resolving powers and that *groEL* genes do not allow a finer strain differentiation [50]. Also, phylogeny based on *imp* gene sequences revealed more phylogenetic divergence within NR-SI-ALY strains than that inferred from *groEL* genes. Distinct lineages were resolved in the phylogenetic tree inferred from *imp* gene sequences, whereas most NR-SI-ALY strains belonged to one clade in the phylogenetic tree inferred from *groEL* gene sequences.

Our RFLP-, sequence-, and phylogeny-based data indicated that genetic differences among NR-SI-ALY strains identified within each gene examined were not related to plant host species, geographical origin, and symptoms shown by infected alder trees. However, they can be the basis for further studies in which other molecular markers should be involved. The most promising approach to find genetic differences among ALY phytoplasma strains linked to pathological and/other biological traits would be sequence comparisons of the entire genome from strains differing greatly in virulence. The results of our study also indicate that ALY phytoplasma is more widespread than previously thought.

## 5. Conclusions

This study updates and extends knowledge on the occurrence, impact, and genetic diversity of ALY phytoplasma in southern Italy. A high percentage of alder trees examined proved to be infected by the ALY phytoplasma. More than half of alder trees that tested phytoplasma-positive did not show clear-cut symptoms. In symptomatic trees, no other cause of disease, such as fungal and/or bacterial infections, could be observed. Therefore, it can be concluded that yellowing, stunting, and decline symptoms exhibited by these trees were incited by the ALY phytoplasma. A considerable genetic variability was observed among the newly recorded ALY phytoplasma strains in southern Italy in almost of the genes examined. However, the genetic differences observed were not related to plant host species, geographical origin, and symptoms shown by infected alder trees. However, they can be the basis for further studies in which additional molecular markers are involved. Also, the results of this study indicate that ALY phytoplasma is more widespread than previously thought.

## Data Availability

DNA sequences generated in this study were deposited in the public GenBank database under accession numbers PP097677 to PP097690 for rRNA, PP125318 to PP125344 for *rpsV* (*rpl22*) and *rpsC* (*rps3*), PP135471 to PP135498 for *map*, PP230419 to PP230443 for *Imp*, and PP230394 to PP230418 for *groEL* genes.

## Supplementary Materials

The following supporting information can be downloaded at: https://www.mdpi.com/article/10.3390/microorganisms12061140/s1, Figure S1: Premature shoots conferring a witches’-broom appearance on common alder trees; Figure S2: *Taq*I and *Bfa*I virtual restriction profiles of rDNA P1A/P7A fragments. Figure S3: Virtual RFLP analysis with 17 restriction enzymes of the 16S rDNA R16F2n/R2 fragments; Figure S4: Phylogenetic tree constructed with 16S rDNA sequences; Figure S5: Phylogenetic tree constructed with 16S/23S rDNA spacer region sequences; Figure S6: Phylogenetic tree constructed with rp genes *rpsV* (*rpl22*) and *rpsC* (*rps3*) sequences; Figure S7: Phylogenetic tree constructed with *map* gene sequences; Table S1: Details of oligonucleotide primers used; Table S2: Phytoplasmas related to the newly recorded alder yellows (ALY) phytoplasma strains in southern Italy examined in this study; Table S3: Summary of the pattern types (subgroups) produced by virtual RFLP analysis of ribosomal protein (rp) gene sequences; Table S4: Levels of 16S rDNA sequence similarity; Table S5: Levels of 16S/23S rDNA spacer region sequence similarity; Table S6: Levels of *rpsV* (*rpl22*) and *rpsC* (*rps3*) gene sequence similarity; Table S7: Levels of *map* gene sequence similarity; Table S8: Summary of the *map* genotypes identified; Table S9: Single nucleotide polymorphisms (SNPs) in the *map* gene sequences; Table S10: Levels of *imp* gene sequence similarity; Table S11: Levels of *groEL* gene sequence similarity.
