# Supplementary material for: Occurrence, Impact, and Multilocus Sequence Analysis of Alder Yellows Phytoplasma Infecting Common Alder and Italian Alder in Southern Italy
Source: Microorganisms. 2024 Jun 4;12(6):1140. doi: 10.3390/microorganisms12061140 (PMC11205446; doi:10.3390/microorganisms12061140)
Supplement: Supplementary file 1 [file microorganisms-12-01140-s001.zip › Supplementary Tables-3a.pdf]

Supplementary Tables

Table S1. Details of oligonucleotide primers used in this study.

Primer	Primer sequence (5'-3')	Sense	Target	References
P1	AGA-GTT-TGA-TCC-TGG-CTC-AGG-A	Forward	16S rDNA	[1]
P7	CGT-CCT-TCA-TCG-GCT-CTT	Reverse	23S rDNA	[1]
P1A	AAC-GCT-GGC-GGC-GCG-CCT-AAT-AC	Forward	16S rDNA	[2]
P7A	CCT-TCA-TCG-GCT-CTT-AGT-GC	Reverse	23S rDNA	[2]
fB1	GAC-CCT-TCA-AAA-GGT-CTT-AG	Forward	16S rDNA	[3]
rULWS	GTC-TTT-TAT-ATA-AGA-GAA-ACA-C	Reverse	16S/23S rDNA spacer region	[3]
R16(V)F1	TTA-AAA-GAC-CTT-CTT-CGG	Forward	16S rDNA	[4]
R16(V)R1	TTC-AAT-CCG-TAC-TGA-GAC-TAC-C	Reverse	16S rDNA	[4]
rp(V)F1	TCG-CGG-TCA-TGC-AAA-AGG-CG	Forward	*rpsS*	[2]
rp(V)R1	ACG-ATA-TTT-AGT-TCT-TTT-TGG	Reverse	*rplP*	[2]
rp(V)F1A	AGG-CGA-TAA-AAA-AGT-TTC-AAA-A	Forward	*rpsS*	[2]
rp(V)R1A	GGC-ATT-AAC-ATA-ATA-TAT-TAT-G	Reverse	*rplP*	[2]
FD9f5	CAA-AAA-ATT-ACT-TTT-GGC-GGG-AC	Forward	*SecY*	[5]
MAPr1	TGC-TCA-AAA-TGA-GCG-CTT-AAA-C	Reverse	*map*	[5]
FD9f6	GTC-GCT-TTA-GAA-TCG-ACA-CA	Forward	*SecY*	[5]
MAPr2	TCG-GAA-GTA-ACA-GCA-GTC-CA	Reverse	*map*	[5]
fEY_imp	CAT-TTT-AAA-TAC-TGT-ATA-TTA-AAT-AC	Forward	*imp*	[6]
rpyrG	GAC-CTT-TTA-AAC-CAC-ATC-C	Reverse	*imp*	[6]
fEY_groEL	GTT-AAT-GAT-GGC-GTT-ACA-ATC-GC	Forward	*groEL*	[6]
rEY_groEL	GTT-AAA-GAA-GGA-CTT-TTA-TCC-GC	Reverse	*groEL*	[6]

Table S2. Phytoplasmas related to the newly recorded alder yellows (ALY) phytoplasma strains in southern Italy examined in this study.

Phytoplasma (strain)	Geographical origin	Sequence*	GenBank accession No.	Reference/collector(s)
Alder yellows (ALY-SI)	Italy	16S, SR	Y16387	[7]
Alder yellows (ALY)	Italy	16S	AY197646	[2]
Alder yellows (ALY882)	Germany	16S	AY197642	[2]
Alder yellows (ALY1068)	Italy	16S	AY197647	[2]
Alder yellows (SW3)	Germany	16S	KP238292	[8]
Alder yellows (SW58)	Germany	16S	KP238298	[8]
Alder yellows (SW1)	Germany	16S	KP238291	[8]
Alder yellows (SW17)	Germany	16S	KP238295	[8]
Alder yellows (ALY-L)	Lithuania	16S	AY028789	[9]
Alder yellows (AldWB)	Poland	16S, SR	MK440303	[10]
Mugwort witches'-broom (MugWB)	Poland	16S, SR	MK440304	[10]
Flavescence dorée (FD70)	France	16S	AY197643	[2]
Flavescence dorée (FD70)	France	16S, SR	AF176319	[11]
Flavescence dorée (FD-C)	Italy	16S	AY197645	[2]
Flavescence dorée (FD-D)	Italy	16S	AY197644	[2]
Flavescence dorée (CH)	Switzerland	16S, SR	CP097583	[12]
Flavescence dorée (FD1487)	Spain	16S, SR	AJ548787	[13]
Flavescence dorée (34c2)	Croatia	16S, SR	HQ712064	[14]
Spartium witches'-broom (SpaWB229)	Italy	16S	AY197652	[2]
Hemp dogbane yellows (HD1)	USA	16S	AY197654	[2]
Virginia creeper (VC)	USA	16S, SR	AF305198	[2]
Rubus stunt (RUS)-Ca.P.rubi	Italy	16S	AY197648	[2]
Rubus stunt (RUS)	Italy	16S, SR	Y16395	[7]
Elm yellows (EY1)-Ca.P.ulmi	USA	16S	AY197655	[2]
Elm yellows (EY1)	USA	16S, SR	AF122910	[15]
Elm yellows (EYCZ1)	Czech Republic	16S, SR	EU184021	[16]
Peach yellows (PY-In)	India	16S	AY197660	[2]
Jujube witches'-broom (JWB-G1)-Ca.P.ziziphi	China	16S	AB052876	[17]
Jujube witches'-broom (JWB)	China	16S	AY197661	[2]
Cherry lethal yellows (CLY-5)	China	16S	AY197659	[2]
Japanese raisin witches'-broom (JRWB)	South Korea	16S, SR	AB442218	[18]
Balanites witches'-broom (BltWB)-Ca.P.balanitae	Myanmar	16S, SR	AB689678	[19]
Alder yellows (ALY)	Italy	*rpsV* (*rpl22*), *rpsC* (*rps3*)	AY197666	[2]
Alder yellows (ALY882)	Germany	*rpsV* (*rpl22*), *rpsC* (*rps3*)	AY197662	[2]
Alder yellows (ALY1068)	Italy	*rpsV* (*rpl22*), *rpsC* (*rps3*)	AY197667	[2]
Alder yellows (AldWB)	Poland	*rpsV* (*rpl22*), *rpsC* (*rps3*)	MK806438	[10]
Mugwort witches'-broom (MugWB)	Poland	*rpsV* (*rpl22*), *rpsC* (*rps3*)	MK814810	[10]
Alder yellows (ALY)	Italy	*rpsV* (*rpl22*), *rpsC* (*rps3*)	JN851860	[20]
Spartium witches'-broom (SpaWB229)	Italy	*rpsV* (*rpl22*), *rpsC* (*rps3*)	AY197672	[2]
Spartium witches'-broom (SpaWB251)	Italy	*rpsV* (*rpl22*), *rpsC* (*rps3*)	AY197673	[2]
Hemp dogbane yellows (HD1)	USA	*rpsV* (*rpl22*), *rpsC* (*rps3*)	AY197674	[2]
Flavescence dorée (FD-C)	Italy	*rpsV* (*rpl22*), *rpsC* (*rps3*)	AY197665	[2]
Flavescence dorée (FD-D)	Italy	*rpsV* (*rpl22*), *rpsC* (*rps3*)	AY197664	[2]
Rubus stunt (RUS)	Italy	*rpsV* (*rpl22*), *rpsC* (*rps3*)	AY197668	[2]
Rubus stunt (RuS400)	Italy	*rpsV* (*rpl22*), *rpsC* (*rps3*)	AY197669	[2]
Rubus stunt (RuS971)	Switzerland	*rpsV* (*rpl22*), *rpsC* (*rps3*)	AY197670	[2]
Rubus stunt (RuSR19)	Germany	*rpsV* (*rpl22*), *rpsC* (*rps3*)	AY197671	[2]
Elm yellows (EY1)	USA	*rpsV* (*rpl22*), *rpsC* (*rps3*)	AY197675	[2]
Elm yellows (EY125)	Italy	*rpsV* (*rpl22*), *rpsC* (*rps3*)	AY197676	[2]
Elm yellows (EY626)	Italy	*rpsV* (*rpl22*), *rpsC* (*rps3*)	AY197677	[2]
Elm yellows (EY627)	Italy	*rpsV* (*rpl22*), *rpsC* (*rps3*)	AY197678	[2]
Elm yellows (NK16)	Croatia	*rpsV* (*rpl22*), *rpsC* (*rps3*)	KU202026	[21]
Elm yellows (EY24-SRB)	Serbia	*rpsV* (*rpl22*), *rpsC* (*rps3*)	HM038464	[22]
Peach yellows (PY-In)	India	*rpsV* (*rpl22*), *rpsC* (*rps3*)	AY197680	[2]
Jujube witches'-broom (JWB)	China	*rpsV* (*rpl22*), *rpsC* (*rps3*)	AY197681	[2]
Cherry lethal yellows (CLY5)	China	*rpsV* (*rpl22*), *rpsC* (*rps3*)	AY197679	[2]
Alder yellows (AI-04-3-13)-M35	Italy	*map*	AM384884	[5]
Alder yellows (74-08-MNE)-M142	Montenegro	*map*	KC188998	[23]
Alder yellows (AI-AL4)-M113	Italy	*map*	LT222008	[24]

Alder yellows (OS-Sep10-179)-M101	Serbia	*map*	LT221996	[24]
Alder yellows (Ag30-MAC)-M149	Macedonia	*map*	KJ605451	[25]
Alder yellows (AI-025-08)-M118	Italy	*map*	LT222013	[24]
Alder yellows (AF-06-30-3)-M50	France	*map*	FN561864	[24]
Alder yellows (AI-040-08)-M55	Italy	*map*	LT221950	[24]
Alder yellows (AF-07-22-30)-M45	France	*map*	LT221940	[24]
Alder yellows (AF-07-28-8)-M31	France	*map*	LT221926	[24]
Alder yellows (AF-07-25-4)-M20	France	*map*	LT221915	[24]
Alder yellows (AS-AL11)-M95	Serbia	*map*	LT221990	[24]
Alder yellows (AF-WJ1444-32)-M2	France	*map*	LT221897	[24]
Alder yellows (SW7)	Germany	*map*	KP238304	[8]
Alder yellows (F14-F6-584)-M123	France	*map*	LR585197	[24]
Alder yellows (G14-F10-224)-M124	Germany	*map*	LR585198	[24]
Alder yellows (AG92)	Bosnia-Herzegovina	*map*	MG551289	Delic D., Radulovic M.
Alder yellows (425)	Croatia	*map*	MG201983	[26]
Alder yellows (85-08-MNE)-M53	Montenegro	*map*	KC189001	[23]
Alder yellows (SW1)	Germany	*map*	KP238302	[8]
Alder yellows (SW38)	Germany	*map*	KP238315	[8]
Alder yellows (SW32)	Germany	*map*	KP238314	[8]
Alder yellows (SW10)	Germany	*map*	KP238306	[8]
Alder yellows (SW13)	Germany	*map*	KP238307	[8]
Alder yellows (SW23)	Germany	*map*	KP238310	[8]
Alderyellows (SW4)	Germany	*map*	KP238303	[8]
Alder yellows (SW8)	Germany	*map*	KP238305	[8]
Alder yellows (SW17)	Germany	*map*	KP238309	[8]
Alder yellows (periwinkle-maintained ALY)-M36	Italy	*map*	AM384885	[5]
Alder yellows (F15-21-153)-M127	France	*map*	LR585201	[24]
Alder yellows (AF-06-30-27)-M48	France	*map*	LT221943	[24]
Alder yellows (75-08-MNE)-M143	Montenegro	*map*	KC188999	[23]
Alder yellows (76-08-MNE)-M43	Montenegro	*map*	KC189000	[23]
Alder yellows (AS-AL11)-M93	Serbia	*map*	LT221988	[24]
Alder yellows (Ag26-MAC)-M146	Macedonia	*map*	KJ605448	[25]
Alder yellows (Ag27-MAC)-M147	Macedonia	*map*	KJ605449	[25]
Alder yellows (Ag29-MAC)-M148	Macedonia	*map*	KJ605450	[25]
Alder yellows (AF-06-30-25)-M37	France	*map*	LT221932	[24]
Alder yellows (AF-07-25-7)-M1	France	*map*	LT221896	[24]
Alder yellows (A06-30-20)	France	*map*	FN561863	[27]
Flavescence dorée (V03-9-2)-M27	France	*map*	AM384888	[5]
Flavescence dorée (V04-11-19)-M34	France	*map*	AM384889	[5]
Flavescence dorée (VI04-188-04)-M12	Italy	*map*	AM384896	[5]
Flavescence dorée (FD70)-M50	France	*map*	AM238512	[5]
Flavescence dorée (VF-09-112)-M36	France	*map*	LT221931	[24]
Flavescence dorée (VF13-380)-M121	France	*map*	LT222016	[24]
Flavescence dorée (V00-SP5)-M54	France	*map*	AM384886	[5]
Flavescence dorée (VF06-30-18)-M54	France	*map*	LT221949	[24]
Flavescence dorée (VI04-Toscana1)-M6	Italy	*map*	AM384895	[5]
Flavescence dorée (VS-Loza-37)-M51	Serbia	*map*	LT221946	[24]
Flavescence dorée (VI04-C28)-M3	Italy	*map*	AM384894	[5]
Flavescence dorée (VI04-188-04)-M12	Italy	*map*	AM384896	[5]
Corylus avellana (FD-D1448)-M159	Slovenia	*map*	OX41712	[28]
Palatinate grapevine yellows (EY17-49)-M53	Germany	*map*	AM384892	[5]
Palatinate grapevine yellows (PGY-B)-M48	Germany	*map*	AM384893	[5]
Palatinate grapevine yellows (EY38)-M46	Germany	*map*	AM384891	[5]
Spartium witches'-broom (SI04-S4)	Italy	*map*	AM384899	[5]
Hemp dogbane yellows (HD1)	USA	*map*	AM384902	[5]
Rubus stunt (RUS)-Ca.P.rubi	Italy	*map*	AM384898	[5]
Elm yellows (EY)-Ca.P.ulmi	France	*map*	AM384901	[5]
Alder yellows (ALY1)	Germany	*imp*	MT668499	[6]
Flavescence dorée (CH)	Switzerland	*imp*	CP097583	[12]
Flavescence dorée (FD-D)	Italy	*imp*	MK614707	[29]
Flavescence dorée (FD-C-Piemonte)	Italy	*imp*	KJ402359	[30]
Flavescence dorée (FD70)	France	*imp*	MT668500	[6]
Elm yellows (4120-Ul-SN)	Germany	*imp*	MT668488	[6]

Elm yellows (1629-Um-BY)	Germany	*imp*	MT668450	[6]
Elm yellows (4157-Ug-SN)	Germany	*imp*	MT668489	[6]
Elm yellows (ULW)	France	*imp*	MT418908	[6]
Jujube witches'-broom (Jwb-nky)	China	*imp*	MG818479	Gao R., Wang J., Lu X.
Alder yellows (periwinkle-maintained ALY)	Italy	*groEL*	MT638097	[6]
Alder yellows (AldY-WA1)	USA	*groEL*	MZ557345	[31]
Elm yellows (6404-Ul-NRW)	Germany	*groEL*	MT638096	[6]
Elm yellows (4120-Ul-SN)	Germany	*groEL*	MT638093	[6]
Elm yellows (ULW)	France	*groEL*	MT418907	[6]
Elm yellows (2906-Ul-ST)	Germany	*groEL*	MT638089	[6]
Elm yellows (3716-Ug-NI)	Germany	*groEL*	MT638090	[6]
Elm yellows (JB0071-C04-CFIA7-2)	France	*groEL*	KJ939992	[32]
Jujube witches'-broom (Jwb-nky)	China	*groEL*	CP025121	[33]
Jujube witches'-broom (Hebei-2018)	China	*groEL*	CP091835	[34]

* 16S, 16S rDNA; SR, 16S/23S rDNA spacer region

Table S3. Summary of the pattern types (subgroups) produced by virtual RFLP analysis of ribosomal protein (rp) gene sequences [rp(V) F1A/rp(V)R1A fragments] of the newly recorded alder yellows (ALY) phytoplasma strains in southern Italy and rp sequences retrieved from the GenBank database. Numbers shown in each column represent distinct RFLP types with each enzyme.

Strain	Genbank Accession. No.	rpV subgroup*	RFLP pattern types								
			*Hpa*II	*Dra*I	*Taq*I	*Alu*I	*Mse*I	*Ssp*I	*Tsp509*I	*Hae*III	*Hha*I
ALY-(ALY)**	AY197666	H	1	1	1	1	1	1	1	1	1
ALY-(ALY882)**	AY197662	K	1	1	1	2	2	2	2	1	1
ALY-(ALY1068)**	AY197667	H	1	1	1	1	1	1	1	1	1
FD-(FD-C)**	AY197665	D	2	1	1	1	2	2	2	1	1
FD-(FD-D)**	AY197664	E	1	1	2	1	3	2	2	1	1
SpaWB-(SpaWB229)**	AY197672	L	1	2	3	1	1	3	3	1	1
ALY-(AldWB)**	MK806438	P[a]	1	1	2	1	2	2	2	1	1
ALY2413	PP125318	H	1	1	1	1	1	1	1	1	1
ALY2436	PP125319	O[a]	1	1	1	1	1	2	2	1	1
ALY2438	PP125320	N[a]	1	1	2	1	1	2	2	1	1
ALY2450	PP125321	S[a]	1	1	1	1	1	1	2	1	1
ALY2454	PP125322	S[a]	1	1	1	1	1	1	2	1	1
ALY2456	PP125323	O[a]	1	1	1	1	1	2	2	1	1
ALY2459	PP125324	H	1	1	1	1	1	1	1	1	1
ALY2460	PP125325	Q[a]	1	1	2	1	1	2	1	1	1
ALY2463	PP125326	N[a]	1	1	2	1	1	2	2	1	1
ALY2465	PP125327	D	2	1	1	1	4	2	2	1	1
ALY2486	PP125328	H	1	1	1	1	1	1	1	1	1
ALY2515	PP125329	S[a]	1	1	1	1	1	1	2	1	1
ALY2518	PP125330	H	1	1	1	1	1	1	1	1	1
ALY2555	PP125331	H	1	1	1	1	1	1	1	1	1
ALY2587	PP125332	R[a]	1	1	1	1	1	2	1	1	1
ALY2588	PP125333	O[a]	1	1	1	1	1	2	2	1	1
ALY2606	PP125334	N[a]	1	1	2	1	1	2	2	1	1
ALY2610	PP125335	N[a]	1	1	2	1	1	2	2	1	1
ALY2611	PP125336	R[a]	1	1	1	1	1	2	1	1	1
ALY2917	PP125337	H	1	1	1	1	1	1	1	1	1
ALY2918	PP125338	H	1	1	1	1	1	1	1	1	1
ALY2921	PP125339	R[a]	1	1	1	1	1	2	1	1	1
ALY2923	PP125340	S[a]	1	1	1	1	1	1	2	1	1
ALY2924	PP125341	H	1	1	1	1	1	1	1	1	1
ALY3016	PP125342	O[a]	1	1	1	1	1	2	2	1	1
ALY3040	PP125343	N[a]	1	1	2	1	1	2	2	1	1
ALY3041	PP125344	H	1	1	1	1	1	1	1	1	1

* rp subgroup designation according to Lee et al. [2] and Martini et al. [35].

** Sequences retrieved from the GenBank database.

[a] Newly identified.

Table S4. Levels of 16S rDNA sequence similarity between the newly recorded alder yellows (ALY) phytoplasma strains in southern Italy.

Strain	ALY2451	ALY2455	ALY2457	ALY2460	ALY2461	ALY2462	ALY2464	ALY2518	ALY2555	ALY2556	ALY2614	ALY2917	ALY2919	ALY2924
ALY2451	100													
ALY2455	98.5	100												
ALY2457	99.0	98.1	100											
ALY2460	99.5	98.6	98.7	100										
ALY2461	99.0	98.1	99.1	98.7	100									
ALY2462	99.5	98.6	98.8	99.2	98.9	100								
ALY2464	99.5	98.7	98.8	99.7	98.8	99.3	100							
ALY2518	99.4	98.3	98.5	99.5	98.5	99.1	99.5	100						
ALY2555	98.3	97.9	97.6	98.5	97.6	98.1	98.4	98.4	100					
ALY2556	98.7	98.1	97.9	98.7	97.9	98.4	98.7	98.8	98.9	100				
ALY2614	99.6	98.7	98.7	99.5	98.7	99.3	99.7	99.3	98.2	98.5	100			
ALY2917	99.7	98.8	99.0	99.4	99.0	99.5	99.5	99.3	98.3	98.6	99.5	100		
ALY2919	99.5	98.6	98.7	99.8	98.7	99.2	99.8	99.5	98.4	98.7	99.7	99.4	100	
ALY2924	98.5	98.2	97.7	98.4	97.7	98.8	99.4	98.3	97.9	98.1	98.2	98.6	98.4	100
ALY-Y16387	99.9	98.9	99.1	99.5	99.1	99.7	99.7	99.4	98.3	98.7	99.6	99.9	99.5	98.5

Table S5. Levels of 16S/23S rDNA spacer region sequence similarity between the newly recorded alder yellows (ALY) phytoplasma strains in southern Italy.

Strain	ALY2451	ALY2455	ALY2457	ALY2460	ALY2461	ALY2462	ALY2464	ALY2518	ALY2555	ALY2556	ALY2614	ALY2917	ALY2919	ALY2924
ALY2451	100													
ALY2455	99.6	100												
ALY2457	95.8	95.4	100											
ALY2460	100	99.6	95.8	100										
ALY2461	93.5	93.2	97.0	93.5	100									
ALY2462	100	99.6	95.8	100	93.5	100								
ALY2464	99.6	99.2	95.4	99.6	93.2	99.6	100							
ALY2518	95.1	94.7	91.6	95.1	89.4	95.1	94.7	100						
ALY2555	98.5	98.1	94.3	98.5	92.0	98.5	98.9	93.5	100					
ALY2556	98.9	98.5	94.7	98.9	92.4	98.9	98.5	93.9	98.9	100				
ALY2614	98.5	98.1	95.1	98.5	92.8	98.5	98.9	93.5	97.7	97.3	100			
ALY2917	92.4	92.0	89.0	92.4	87.8	92.4	92.0	87.5	90.9	91.3	90.9	100		
ALY2919	99.6	99.2	95.4	99.6	93.2	99.6	100	94.7	98.9	98.5	98.9	92.0	100	
ALY2924	98.1	97.7	96.6	98.1	94.3	98.1	97.7	94.7	96.6	97.0	96.6	90.5	97.7	100
ALY-Y16387	99.2	98.9	95.1	99.2	92.8	99.2	98.9	94.3	97.7	98.1	97.7	91.6	98.9	97.3

Table S6. Levels of *rpsV* (*rpl22*) and *rpsC* (*rps3*) gene sequence similarity between the newly recorded alder yellows (ALY) phytoplasma strains in southern Italy.

Strain	ALY2413	ALY2436	ALY2438	ALY2450	ALY2454	ALY2456	ALY2459	ALY2460	ALY2463	ALY2465	ALY2486	ALY2515	ALY2518	ALY2555	ALY2587	ALY2588	ALY2606	ALY2610	ALY2611	ALY2917	ALY2918	ALY2921	ALY2923	ALY2924	ALY3016	ALY3040	ALY3041
ALY2413	100																										
ALY2436	99.6	100																									
ALY2438	99.5	99.6	100																								
ALY2450	99.7	99.7	99.6	100																							
ALY2454	99.8	99.8	99.6	99.9	100																						
ALY2456	99.6	99.8	99.8	99.7	99.8	100																					
ALY2459	99.9	99.7	99.6	99.8	99.9	99.7	100																				
ALY2460	99.6	99.6	99.6	99.6	99.6	99.6	99.7	100																			
ALY2463	99.4	99.6	99.9	99.5	99.6	99.7	99.5	99.6	100																		
ALY2465	99.0	99.2	99.2	99.1	99.2	99.2	99.1	99.0	99.1	100																	
ALY2486	99.6	99.5	99.1	99.4	99.5	99.3	99.6	99.3	99.0	98.8	100																
ALY2515	99.6	99.6	99.5	99.7	99.8	99.6	99.7	99.5	99.4	99.2	99.5	100															
ALY2518	99.9	99.6	99.4	99.6	99.7	99.6	99.8	99.6	99.3	99.1	99.7	99.7	100														
ALY2555	99.9	99.6	99.4	99.6	99.7	99.6	99.8	99.6	99.3	99.1	99.7	99.7	100	100													
ALY2587	99.6	99.7	99.6	99.5	99.6	99.6	99.6	99.6	99.5	99.3	99.6	99.6	99.6	99.6	100												
ALY2588	99.3	99.5	99.3	99.4	99.5	99.5	99.4	99.3	99.2	99.0	99.1	99.5	99.4	99.4	99.4	100											
ALY2606	99.2	99.4	99.6	99.5	99.4	99.4	99.3	99.4	99.5	99.1	99.0	99.4	99.3	99.3	99.5	99.2	100										
ALY2610	99.2	99.6	99.7	99.3	99.4	99.6	99.3	99.6	99.6	99.1	99.2	99.4	99.3	99.3	99.6	99.2	99.5	100									
ALY2611	99.8	99.6	99.5	99.6	99.6	99.6	99.7	99.6	99.4	99.2	99.6	99.6	99.9	99.9	99.7	99.5	99.4	99.4	100								
ALY2917	99.8	99.6	99.3	99.6	99.6	99.5	99.7	99.5	99.2	99.0	99.8	99.6	99.9	99.9	99.7	99.3	99.5	99.4	99.8	100							
ALY2918	99.8	99.6	99.3	99.6	99.6	99.5	99.7	99.5	99.2	99.0	99.8	99.6	99.9	99.9	99.7	99.3	99.2	99.4	99.8	100	100						
ALY2921	99.6	99.6	99.3	99.4	99.5	99.5	99.6	99.5	99.2	99.0	99.6	99.5	99.7	99.7	99.7	99.3	99.2	99.4	99.8	99.8	99.8	100					
ALY2923	99.6	99.6	99.5	99.9	99.8	99.6	99.7	99.5	99.4	99.2	99.5	99.8	99.7	99.7	99.6	99.5	99.6	99.4	99.6	99.6	99.6	99.5	100				
ALY2924	99.6	99.6	99.3	99.6	99.6	99.5	99.7	99.5	99.2	99.2	99.6	99.6	99.7	99.7	99.7	99.3	99.2	99.4	99.6	99.8	99.8	99.6	99.6	100			
ALY3016	99.3	99.5	99.5	99.4	99.5	99.5	99.4	99.5	99.4	99.4	99.1	99.5	99.4	99.4	99.6	99.3	99.4	99.6	99.5	99.3	99.3	99.3	99.5	99.5	100		
ALY3040	99.2	99.2	99.6	99.3	99.2	99.4	99.1	99.2	99.1	99.1	99.0	99.2	99.3	99.3	99.3	99.0	99.5	99.5	99.4	99.2	99.2	99.2	99.4	99.2	99.4	100	
ALY3041	99.7	99.6	99.2	99.5	99.6	99.4	99.6	99.4	99.1	99.1	99.0	99.6	99.8	99.8	99.6	99.2	99.1	99.3	99.7	99.9	99.9	99.7	99.6	99.2	99.4	99.3	100
ALY-AY197666	99.8	99.6	99.3	99.6	99.6	99.5	99.7	99.5	99.0	99.0	99.0	99.5	99.7	99.7	99.6	99.1	99	99.2	99.6	99.8	99.8	99.6	99.5	99.8	99.3	99.2	99.9

Table S7. Levels of *map* gene sequence similarity between the newly recorded alder yellows (ALY) phytoplasma strains in southern Italy.

Strain	ALY2514	ALY2515	ALY2587	ALY2590	ALY2599	ALY2600	ALY2603	ALY2604	ALY2610	ALY2687	ALY2689	ALY2711	ALY2712	ALY2735	ALY2745	ALY2912	ALY2917	ALY2919	ALY2921	ALY2924	ALY3014	ALY3015	ALY3016	ALY3018	ALY3021	ALY3040	ALY3041	ALY3043
ALY2514	100																											
ALY2515	99.5	100																										
ALY2587	99.5	100	100																									
ALY2590	99.8	99.4	99.4	100																								
ALY2599	99.5	100	100	99.4	100																							
ALY2600	99.5	100	100	99.4	100	100																						
ALY2603	99.5	100	100	99.4	100	100	100																					
ALY2604	99.7	99.8	99.8	99.5	99.8	99.8	99.8	100																				
ALY2610	99.4	99.8	99.8	99.2	99.8	99.8	99.8	99.7	100																			
ALY2687	99.2	99.1	99.1	99.1	99.1	99.1	99.1	99.2	98.9	100																		
ALY2689	99.5	100	100	99.4	100	100	100	99.8	99.8	99.1	100																	
ALY2711	99.1	99.5	99.5	98.9	99.5	99.5	99.5	99.4	99.4	98.6	99.5	100																
ALY2712	99.4	99.8	99.8	99.2	99.8	99.8	99.8	99.7	99.7	98.9	99.8	99.4	100															
ALY2735	99.5	100	100	99.4	100	100	100	99.8	99.8	99.1	100	99.5	99.8	100														
ALY2745	99.4	99.8	99.8	99.2	99.8	99.8	99.8	99.7	99.7	98.9	99.8	99.4	99.7	99.8	100													
ALY2912	99.4	99.8	99.8	99.2	99.8	99.8	99.8	99.7	99.7	98.9	99.8	99.4	99.7	99.8	99.7	100												
ALY2917	99.8	99.7	99.7	99.7	99.7	99.7	99.7	99.8	99.5	99.1	99.7	99.2	99.5	99.7	99.5	99.5	100											
ALY2919	100	99.5	99.5	99.8	99.5	99.5	99.5	99.7	99.4	99.2	99.5	99.1	99.4	99.5	99.4	99.4	99.8	100										
ALY2921	100	99.5	99.5	99.8	99.5	99.5	99.5	99.7	99.4	99.2	99.5	99.1	99.4	99.5	99.4	99.4	99.8	100	100									
ALY2924	99.5	100	100	99.4	100	100	100	99.8	99.8	99.1	100	99.5	99.8	100	99.8	99.8	99.7	99.5	99.5	100								
ALY3014	99.5	100	100	99.4	100	100	100	99.8	99.8	99.1	100	99.5	99.8	100	99.8	99.8	99.7	99.5	99.5	100	100							
ALY3015	99.5	100	100	99.4	100	100	100	99.8	99.8	99.1	100	99.5	99.8	100	99.8	99.8	99.7	99.5	99.5	100	100	100						
ALY3016	99.7	99.8	99.8	99.5	99.8	99.8	99.8	99.7	99.7	98.9	99.8	99.4	99.7	99.8	99.7	99.7	99.8	99.5	99.7	99.8	99.8	99.8	100					
ALY3018	99.5	100	100	99.4	100	100	100	99.8	99.8	99.1	100	99.5	99.8	100	99.8	99.8	99.7	99.5	99.5	100	100	100	99.8	100				
ALY3021	99.5	100	100	99.4	100	100	100	99.8	99.8	99.1	100	99.5	99.8	100	99.8	99.8	99.7	99.5	99.5	100	100	100	99.8	100	100			
ALY3040	99.5	100	100	99.4	100	100	100	99.8	99.8	99.1	100	99.5	99.8	100	99.8	99.8	99.7	99.5	99.5	100	100	100	99.8	100	100	100		
ALY3041	99.8	99.7	99.7	99.7	99.7	99.7	99.7	99.8	99.5	99.1	99.7	99.2	99.5	99.7	99.5	99.5	100	99.8	99.8	99.7	99.7	99.7	99.8	99.7	99.7	99.7	100	
ALY3043	99.2	99.1	99.1	99.1	99.1	99.1	99.1	99.2	98.9	99.4	99.1	98.6	99.2	99.1	98.9	98.9	99.1	99.2	99.2	99.1	99.1	99.1	98.9	99.1	99.1	99.1	99.1	
ALY-M36-AM384885	99.2	99.1	99.1	99.1	99.1	99.1	99.1	99.2	98.9	99.4	99.1	98.6	99.2	99.1	98.9	98.9	99.1	99.2	99.2	99.1	99.1	99.1	98.9	99.1	99.1	99.1	99.1	100

Table S8. Summary of the *map* genotypes identified in the newly recorded alder yellows (ALY) phytoplasma strains in southern Italy.

Strain	Genbank Accession. No.	Host	*Map* genotype*
ALY2514	PP135471	*Alnus glutinosa*	M113
ALY2515	PP135472	*Alnus glutinosa*	M163[a]
ALY2587	PP135473	*Alnus cordata*	M163[a]
ALY2590	PP135474	*Alnus cordata*	M164[a]
ALY2599	PP135475	*Alnus glutinosa*	M163[a]
ALY2600	PP135476	*Alnus glutinosa*	M163[a]
ALY2603	PP135477	*Alnus glutinosa*	M163[a]
ALY2604	PP135478	*Alnus glutinosa*	M165[a]
ALY2610	PP135479	*Alnus cordata*	M166[a]
ALY2687	PP135480	*Alnus cordata*	M167[a]
ALY2689	PP135481	*Alnus glutinosa*	M163[a]
ALY2711	PP135482	*Alnus glutinosa*	M168[a]
ALY2712	PP135483	*Alnus glutinosa*	M169[a]
ALY2735	PP135484	*Alnus glutinosa*	M163[a]
ALY2745	PP135485	*Alnus glutinosa*	M170[a]
ALY2912	PP135486	*Alnus glutinosa*	M171[a]
ALY2917	PP135487	*Alnus glutinosa*	M173[a]
ALY2919	PP135488	*Alnus glutinosa*	M113
ALY2921	PP135489	*Alnus cordata*	M113
ALY2924	PP135490	*Alnus glutinosa*	M163[a]
ALY3014	PP135491	*Alnus glutinosa*	M163[a]
ALY3015	PP135492	*Alnus cordata*	M163[a]
ALY3016	PP135493	*Alnus glutinosa*	M172[a]
ALY3018	PP135494	*Alnus glutinosa*	M163[a]
ALY3021	PP135495	*Alnus cordata*	M163[a]
ALY3040	PP135496	*Alnus cordata*	M163[a]
ALY3041	PP135497	*Alnus glutinosa*	M173[a]
ALY3043	PP135498	*Alnus glutinosa*	M36

*. *Map* genotype designation according to Arnaud et al. [5]; Malembic-Maher et al. [24]; Zwitter et al., [28]; Krstić et al. [36]; Rigamonti et al., [37].

[a]. Newly identified.

Table S9. Single nucleotide polymorphisms (SNPs) in the *map* gene sequences of newly recorded alder yellows (ALY) phytoplasma strains in southern Italy and reference phytoplasma strains.

Strain	Genbank Acc. No.	*Map* Genotype*	SNP position											
			11	166	195	216	226	313	372	398	404	456	593	604
ALY-(AI-04-3-13)	AM384884	M35	G	A	G	G	C	C	G	A	G	C	A	C
ALY-(AI-AL4)	LT222008	M113	G	G	C	G	C	C	G	A	G	C	C	C
ALY-(74-08-MNE)	KC188998	M142	G	G	G	G	C	C	G	A	G	C	C	C
FD-D1448	OX417124	M159	G	G	G	G	C	C	G	A	G	C	C	A
ALY	AM384885	M36	G	G	G	G	C	C	G	A	G	A	C	C
ALY2514	PP135471	M113	G	G	C	G	C	C	G	A	G	C	C	C
ALY2515	PP135472	M163[a]	G	A	G	G	C	C	G	A	G	C	C	C
ALY2587	PP135473	M163[a]	G	A	G	G	C	C	G	A	G	C	C	C
ALY2590	PP135474	M164[a]	G	G	C	G	C	C	A	A	A	C	C	C
ALY2599	PP135475	M163[a]	G	A	G	G	C	C	G	A	G	C	C	C
ALY2600	PP135476	M163[a]	G	A	G	G	C	C	G	A	G	C	C	C
ALY2603	PP135477	M163[a]	G	A	G	G	C	C	G	A	G	C	C	C
ALY2604	PP135478	M165[a]	G	A	G	G	C	C	G	A	G	C	C	C
ALY2610	PP135479	M166[a]	G	A	G	G	C	C	T	A	G	C	C	C
ALY2687	PP135480	M167[a]	G	G	G	T	C	T	G	A	G	C	C	C
ALY2689	PP135481	M163[a]	G	A	G	G	C	C	G	A	G	C	C	C
ALY2711	PP135482	M168[a]	A	A	G	G	C	C	G	A	G	C	C	C
ALY2712	PP135483	M169[a]	G	A	G	G	C	C	G	A	G	A	C	C
ALY2735	PP135484	M163[a]	G	A	G	G	C	C	G	A	G	C	C	C
ALY2745	PP135485	M170[a]	G	A	G	G	C	C	G	C	G	C	C	C
ALY2912	PP135486	M171[a]	G	A	G	G	T	C	G	A	G	C	C	C
ALY2917	PP135487	M173[a]	G	A	C	G	C	C	G	A	G	C	C	C
ALY2919	PP135488	M113	G	G	C	G	C	C	G	A	G	C	C	C
ALY2921	PP135489	M113	G	G	C	G	C	C	G	A	G	C	C	C
ALY2924	PP135490	M163[a]	G	A	G	G	C	C	G	A	G	C	C	C
ALY3014	PP135491	M163[a]	G	A	G	G	C	C	G	A	G	C	C	C
ALY3015	PP135492	M163[a]	G	A	G	G	C	C	G	A	G	C	C	C
ALY3016	PP135493	M172[a]	G	A	C	G	C	C	G	A	G	C	C	C
ALY3018	PP135494	M163[a]	G	A	G	G	C	C	G	A	G	C	C	C
ALY3021	PP135495	M163[a]	G	A	G	G	C	C	G	A	G	C	C	C
ALY3040	PP135496	M163[a]	G	A	G	G	C	C	G	A	G	C	C	C
ALY3041	PP135497	M173[a]	G	A	C	G	C	C	G	A	G	C	C	C
ALY3043	PP135498	M36	G	G	G	G	C	C	G	A	G	A	C	C

*. *Map* genotype designation according to Arnaud et al. [25]; Malembic-Maher et al. [24]; Zwitter et al., [28]; Krstić et al. [36]; Rigamonti et al., [37].
[a.] Newly identified.

Table S10. Levels of *Imp* gene sequence similarity between the newly recorded alder yellows (ALY) phytoplasma strains in southern Italy.

Strain	ALY2453	ALY2462	ALY2585	ALY2588	ALY2600	ALY2603	ALY2604	ALY2605	ALY2606	ALY2609	ALY2610	ALY2611	ALY2612	ALY2614	ALY2917	ALY2919	ALY2921	ALY2923	ALY2924	ALY3014	ALY3015	ALY3016	ALY3017	ALY3019	ALY3021
ALY2453	100																								
ALY2462	94.5	100																							
ALY2585	94.1	98.4	100																						
ALY2588	81.0	84.4	83.8	100																					
ALY2600	80.0	83.6	83.6	94.3	100																				
ALY2603	71.7	74.9	74.5	76.4	76.0	100																			
ALY2604	93.7	99.0	97.4	83.8	83.4	75.8	100																		
ALY2605	90.3	95.2	95.2	83.4	83.0	77.6	95.0	100																	
ALY2606	94.5	100	98.4	84.4	83.6	74.9	99.0	95.2	100																
ALY2609	92.5	97.2	96.0	82.6	82.2	74.5	96.2	93.3	97.2	100															
ALY2610	79.4	82.4	82.4	82.3	95.6	75.0	82.2	82.0	82.4	81.4	100														
ALY2611	93.5	99.0	97.8	83.8	83.4	74.7	98.4	95.0	99.0	96.2	82.2	100													
ALY2612	93.7	99.2	97.6	84.0	83.2	74.5	98.2	94.5	99.2	96.8	82.0	98.2	100												
ALY2614	93.9	99.0	97.4	84.4	83.2	74.5	98.0	94.5	99.0	96.6	82.0	98.0	99.0	100											
ALY2917	93.5	98.8	97.6	83.6	84.0	74.5	97.8	94.7	98.0	95.6	82.8	97.8	97.2	97.0	100										
ALY2919	92.7	98.2	97.4	84.6	85.0	75.2	97.6	94.3	98.2	95.8	83.8	98.0	97.4	97.2	97.4	100									
ALY2921	81.6	84.6	84.4	93.9	96.8	76.2	84.4	84.0	84.6	83.2	95.4	84.4	84.2	84.2	85.0	85.9	100								
ALY2923	80.2	84.2	84.2	92.7	95.0	75.4	84.0	84.0	84.2	83.2	93.5	84.0	83.8	83.8	84.2	85.1	94.5	100							
ALY2924	78.6	83.0	83.4	92.3	91.1	77.4	82.8	83.0	83.0	81.8	90.3	82.8	82.6	82.6	83.0	84.4	91.5	90.9	100						
ALY3014	92.5	97.6	96.4	83.4	83.2	76.4	97.0	94.1	97.6	94.9	82.0	97.0	96.8	96.6	96.2	96.2	84.2	83.6	83.4	100					
ALY3015	79.0	82.4	82.6	94.9	92.7	76.5	81.8	82.4	82.4	81.2	91.7	81.8	82.0	82.8	82.0	83.0	91.9	92.7	90.1	81.4	100				
ALY3016	73.1	76.2	76.6	77.4	76.4	91.8	77.2	80.0	76.2	75.4	75.0	76.0	75.6	76.0	75.6	76.8	76.4	76.4	78.2	77.8	76.0	100			
ALY3017	70.9	73.2	73.9	76.6	75.6	93.6	74.7	77.8	73.7	73.1	74.3	73.5	73.3	73.7	73.3	74.5	75.6	75.6	77.2	75.2	75.2	96.6	100		
ALY3019	93.3	98.0	96.8	83.0	82.4	73.7	97.0	94.1	98.0	95.2	81.0	97.0	97.2	97.0	96.0	96.2	83.2	82.8	81.6	95.6	81.0	75.6	73.1	100	
ALY3021	72.7	75.0	75.0	75.6	76.0	97.1	75.2	78.2	75.0	74.7	75.0	74.5	74.7	74.7	74.3	75.0	76.2	75.4	76.2	76.2	74.1	90.1	91.6	74.3	100
ALY1-MT668499	94.3	99.8	98.2	84.2	83.4	74.7	98.8	95.0	99.8	97.0	82.0	98.8	99.0	98.8	97.8	98.0	84.4	84.0	82.8	97.4	82.2	76.0	73.5	97.8	74.9

Table S11. Levels of *groEL* gene sequence similarity between the newly recorded alder yellows (ALY) phytoplasma strains in southern Italy.

Strain	ALY2414	ALY2453	ALY2462	ALY2585	ALY2588	ALY2600	ALY2603	ALY2604	ALY2605	ALY2606	ALY2609	ALY2610	ALY2611	ALY2612	ALY2917	ALY2919	ALY2921	ALY2923	ALY2924	ALY3014	ALY3015	ALY3016	ALY3017	ALY3019	ALY3021
ALY2414	100																								
ALY2453	98.7	100																							
ALY2462	99.5	98.2	100																						
ALY2585	99.9	98.6	99.6	100																					
ALY2588	99.7	98.7	99.5	99.9	100																				
ALY2600	99.6	98.3	99.9	99.7	99.6	100																			
ALY2603	99.7	98.5	99.5	99.9	99.7	99.6	100																		
ALY2604	99.6	98.3	99.4	99.7	99.6	99.5	99.6	100																	
ALY2605	99.6	98.6	99.4	99.7	99.9	99.5	99.6	99.5	100																
ALY2606	99.6	98.6	98.4	99.7	99.9	99.5	99.6	99.5	100	100															
ALY2609	99.9	98.6	99.4	99.7	99.9	99.5	99.6	99.5	100	100	100														
ALY2610	99.5	98.6	99.4	100	99.9	99.7	99.9	99.7	99.7	99.7	99.7	100													
ALY2611	99.5	98.5	99.5	99.6	99.7	99.4	99.5	99.4	99.9	99.9	99.9	99.6	100												
ALY2612	99.7	98.5	99.5	99.9	99.7	99.6	99.7	99.6	99.9	99.9	99.9	99.9	99.7	100											
ALY2917	99.7	98.5	99.5	99.9	99.7	99.6	99.7	99.6	99.9	99.9	99.9	99.9	99.7	100	100										
ALY2919	99.9	98.6	99.6	100	99.9	99.7	99.9	99.7	99.7	99.7	99.7	100	99.6	99.9	99.9	100									
ALY2921	99.5	98.5	99.2	99.6	99.7	99.4	99.5	99.6	99.9	99.9	99.9	99.6	99.7	99.7	99.7	99.6	100								
ALY2923	99.4	98.1	99.4	99.2	99.1	99.2	99.1	99.0	99.2	99.2	99.2	99.2	99.4	99.4	99.4	99.2	99.1	100							
ALY2924	99.6	98.3	99.4	99.7	99.6	99.5	99.6	99.5	99.7	99.7	99.7	99.7	99.6	99.9	99.9	99.7	99.6	99.2	100						
ALY3014	99.5	98.5	99.7	99.6	99.7	99.6	99.5	99.4	99.6	99.6	99.6	99.6	99.7	99.5	99.5	99.6	99.5	99.4	99.4	100					
ALY3015	99.7	98.5	99.5	99.9	99.7	99.6	99.7	99.6	99.9	99.9	99.9	99.9	99.7	100	100	99.9	99.7	99.4	99.9	99.5	100				
ALY3016	99.7	98.7	99.5	99.9	100	99.6	99.7	99.6	99.9	99.9	99.9	99.9	99.7	99.7	99.7	99.9	99.7	99.1	99.6	99.7	99.7	100			
ALY3017	99.6	98.6	99.4	99.7	99.9	99.5	99.6	99.5	100	100	100	99.7	99.9	99.5	99.9	99.7	99.9	99.2	99.7	99.6	99.9	99.9	100		
ALY3019	99.1	98.1	99.8	99.2	99.4	99.0	99.1	99.2	99.2	99.2	99.2	99.2	99.1	99.1	99.1	99.2	99.1	98.2	99.0	99.1	99.1	99.4	99.2	100	
ALY3021	99.7	98.7	99.5	99.9	100	99.6	99.7	99.6	99.9	99.9	99.9	99.9	99.7	99.7	99.7	99.9	99.7	98.1	99.6	99.7	99.7	100	99.9	99.4	100
ALY-MT638097	99.0	97.7	99.2	98.8	98.7	99.1	98.7	98.6	98.8	98.8	98.8	98.8	99.0	99.0	99	98.8	98.7	98.6	98.8	99	99	98.7	98.8	98.1	98.7

References

1. Schneider, B.; Seemüller, E.; Smart, C.D.; Kirkpatrick, B.C. Phylogenetic classification of plant pathogenic mycoplasma-like organisms or phytoplasmas. In *Molecular and diagnostic procedures in mycoplasmology*; Razin, S., Tully, J.G. Eds.; Academic Press: San Diego, California, USA, 1995; Volume I, pp. 369-380.
2. Lee, I.-M.; Martini, M.; Marcone, C.; Zhu, S.F. Classification of phytoplasma strains in the elm yellows group (16SrV) and proposal of '*Candidatus* Phytoplasma ulmi' for the phytoplasma associated with elm yellows. *Int. J. Syst. Evol. Microbiol*. **2004**, 54, 337-347.
3. Smart, C.D.; Schneider, B.; Blomquist, C.L.; Guerra, L.J.; Harrison, N.A.; Ahrens, U.; Lorenz, K.-H.; Seemüller, E.; Kirkpatrick, B.C. Phytoplasma-specific PCR primers based on sequences of the 16S/23S rRNA spacer region. *Appl. Environ. Microbiol*. **1996**, 62, 2988-2993.
4. Lee, I.-M.; Gundersen, D.E.; Hammond, R.W.; Davis, R.E. Use of mycoplasmalike organism (MLO) group-specific oligonucleotide primers for nested-PCR assays to detect mixed-MLO infections in a single host plant. *Phytopathology* **1994**, 84, 559-566.
5. Arnaud, G.; Malembic-Maher, S.; Salar, P.; Bonnet, P.; Maixner, M.; Marcone, C.; Boudon-Padieu, E.; Foissac, X. Multilocus sequence typing confirms the close genetic interrelatedness of three distinct flavescence dorée phytoplasma strain clusters and group 16SrV phytoplasmas infecting grapevine and alder in Europe. *Appl. Environ. Microbiol*. **2007**, 73, 4001-4010.
6. Schneider, B.; Hüttel, B.; Zübert, C.; Kube, M. Genetic variation, phylogenetic relationship and spatial distribution of *'Candidatus* Phytoplasma ulmi' strains in Germany. *Sci. Rep*. **2020**, 10, 21864.
7. Seemüller, E.; Marcone, C.; Lauer, U.; Ragozzino, A.; Göschl, M. Current status of molecular classification of the phytoplasmas. *J. Plant Pathol.* **1998**, 80, 3-26.
8. Holz, S.; Duduk, B.; Büttner, C.; Kube, M. Genetic variability of alder yellows phytoplasma in *Alnus glutinosa* in its natural Spreewald habitat. *For. Pathol.* **2016**, 46, 11-21.

9. Valiunas, D.; Alminaite, A.; Staniulis, J.; Jomantiene, R.; Davis, R.E. First report of alder yellows phytoplasma in the eastern Baltic Region. *Plant Dis.* **2001**, 85, 1120.
10. Jurga, M.; Zwolińska, A. *Artemisia vulgaris*, a new host of 16SrV-C phytoplasma related strains infecting black alder in Poland. *J. Phytopathol.* **2020**, 168, 659-667.
11. Davis, R.E.; Dally, E.L. Revised subgroup classification of group 16SrV phytoplasmas and placement of Flavescence dorée eassociated phytoplasmas in two distinct subgroups. *Plant Dis.* **2001**, 85, 790–797.
12. Debonneville, C.; Mandelli, L.; Brodard, J.; Groux, R.; Roquis, D.; Schumpp, O. The complete genome of the "Flavescence dorée" phytoplasma reveals characteristics of low genome plasticity. *Biology* **2022**, 11, 95.
13. Bertaccini, A., Lee, I.-M. Phytoplasmas: an update. In *Phytoplasmas: Plant Pathogenic Bacteria-I. Characterization and Epidemiology of Phytoplasma-Associated Diseases*; Rao, G.P., Bertaccini, A., Fiore, N., Liefting, L., Eds.; Springer Nature: Singapore, 2018; pp. 1-29.
14. Šeruga Musić, M.; Škorić, D.; Haluška, I.; Križanac, I.; Plavec, J.; Mikec, I. First report of Flavescence dorée-related phytoplasma affecting grapevines in Croatia. *Plant Dis.* **2011**, 95(3) 353.
15. Griffiths, H.M.; Sinclair, W.A.; Boudon-Padieu, E.; Daire, X.; Lee, I.-M.; Sfalanga, A; Bertaccini, A. Phytoplasmas associated with elm yellows: molecular variability and differentiation from related organisms. Plant Dis. **1999**, 83, 1101–1104.
16. Navrátil, M.; Šafářová, D.; Válová, P.; Fránová, J.; Šimková, M. Phytoplasma associated with witches'-broom disease of *Ulmus minor* Mill. in the Czech Republic: electron miscoscopy and molecular characterization. *Folia Microbiol*. **2009**, 54, 37-42.
17. Jung, H.-Y.; Sawayanagi, T.; Kakizawa, S.; Nishigawa, H.; Wei, W.; Oshima, K.; Miyata, S.-i.; Ugaki, M.; Hibi, T.; Namba S. '*Candidatus* Phytoplasma ziziphi', a novel phytoplasma taxon associated with jujube witches'-broom disease. *Int. J. Syst. Evol. Microbiol*. **2003**, 53, 1037-1041.
18. Kamala-Kannan, S.; Han, S.-S.; Lee, K.-J.; Velmurugan, P.; Lee, Y.H.; Chae, J.-C.; Lee, Y.-S.; Lee, J.-Y.; Oh, B.-T. Association of elm yellows subgroup 16SrV-B phytoplasma with a disease of *Hovenia dulcis*. *J. Phytopathol.* **2011**, 159, 171-174.
19. Win, N.K.K.; Lee, S.-Y.; Bertaccini, A.; Namba, S.; Jung, H.-Y. '*Candidatus* Phytoplasma balanitae' associated with witches' broom disease of *Balanites triflora*. *Int. J. Syst. Evol. Microbiol*. **2013**, 63**,** 636-640.
20. Durante, G.; Casati, P.; Clair, D.; Quaglino, F.; Bulgari, D.; Boudon-Padieu, E.; Bianco, P.A. Sequence analyses of S10-*spc* operon among 16SrV group phytoplasmas: phylogenetic relationships and identification of discriminating single nucleotide polymorphisms. *Ann. Appl. Biol*. **2012**, 161, 234–246.
21. Katanić, Z.; Krstin, L.; Ježić, M.; Zebec, M; Ćurković-Perica, M. Molecular characterization of elm yellows phytoplasmas in Croatia and their impact on *Ulmus* spp. *Plant Pathol*. **2016**, 65, 1430-1440.
22. Jović, J.; Cvrković, T.; Mitrović, M.; Petrović, A.; Krstić, O.; Krnjajić, S.; Toševski, I. Multigene sequence data and genetic diversity among '*Candidatus* Phytoplasma ulmi' strains infecting *Ulmus* spp. in Serbia. *Plant Pathol*. **2011**, 60, 356-368.
23. Radonjić, S.; Hrnčic, S.; Krstić, O.; Cvrković, T.; Mitrović, M.; Jović, J.; Toševski, I. First report of alder yellows phytoplasma infecting common and grey alder (*Alnus glutinosa* and *A. incana*) in Montenegro. *Plant Dis*. **2013**, 97, 686.
24. Malembic-Maher, S.; Desqué, D.; Khalil, D.; Salar, P.; Bergey, B.; Danet, J.-L.; Duret, S.; Dubrana-Ourabah, M.-P.; Beven, L.; Ember, I.; Acs, Z.; Della Bartola, M.; Materazzi, A.; Filippin, L.; Krnjajic, S.; Krstić, O.; Toševski, I.; Lang, F.; Jarausch, B., Kölber, M.; Jović, J.; Angelini, E.; Arricau-Bouvery, N.; Maixner, M.; Foissac, X. When a Palearctic bacterium meets a Nearctic insect vector: Genetic and ecological insights into the emergence of the grapevine Flavescence doreé epidemics in Europe. *PLoS Pathog.* **2020**, 16(3): e1007967.
25. Atanasova, B.; Spasov, D., Jakovljević, M.; Jović, J.; Krstić, O.; Mitrović, M.; Cvrković, T. First report of alder yellows phytoplasma associated with common alder (*Alnus glutinosa*) in the Republic of Macedonia. *Plant Dis*. **2014**, 98, 1268.
26. Plavec, J.; Budinšćak, Ž.; Križanac, I.; Škorić, D.; Foissac, X.; Šeruga Musić, M. Multilocus sequence typing reveals the presence of three distinct flavescence dorée phytoplasma genetic clusters in Croatian vineyards. *Plant Pathol*. **2019**, 68, 18–30.
27. Malembic-Maher, S.; Salar, P.; Filippin, L.; Carle, P.; Angelini, E.; Foissac, X. Genetic diversity of European phytoplasmas of the 16SrV taxonomic group and proposal of '*Candidatus* Phytoplasma rubi'. *Int. J. Syst. Evol. Microbiol*. **2011**, 61, 2129-2134.
28. Zwitter Z.K.; Seljak, G.; Jakomin, T.; Brodarić, J.; Vućurović, A.; Pedemay, S.; Salar, P.; Malembic-Maher, S.; Foissac, X.; Mehle, N. Epidemiology of flavescence dorée and hazelnut decline in Slovenia: geographical distribution and genetic diversity of the associated 16SrV phytoplasmas. Front. Plant Sci. 2023, 14, 1217425.
29. Trivellone, V.; Ripamonti, M.; Angelini, E.; Filippin, L.; Rossi, M.; Marzachì, C. Galetto, L. Evidence suggesting interactions between immunodominant membrane protein Imp of Flavescence dorée phytoplasma and protein extracts from distantly related insect species. *J. Appl. Microbiol.* **2019,** 127(6), 1801–1813.
30. Rashidi, M.; Galetto, L.; Bosco, D.; Bulgarelli, A.; Vallino, M.; Veratti, F.; Marzachì, C. Role of the major antigenic membrane protein in phytoplasma transmission by two insect vector species. *BMC Microbiol.* **2015**, 15, 193.
31. Cai, W.; Nunziata, S.O.; Srivastava, S.K.; Wilson, T.; Chambers, N.; Rivera, Y.; Nakhla, M.; Costanzo, S. Draft genome sequence resource of AldY-WA1, a phytoplasma strain associated with alder yellows of *Alnus rubra* in Washington, U.S.A. *Plant Dis*. **2022**, 106, 1971-1973.

32. Dumonceaux, T.J.; Green, M.; Hammond, C.; Perez, E; Olivier, C. Molecular diagnostic tools for detection and differentiation of phytoplasmas based on chaperonin-60 reveal differences in host plant infection patterns. *PloS ONE* **2014**, 9(12), e116039.
33. Wang, J.; Song, L.; Jiao, Q.; Yang, S.; Gao, R.; Lu, X.; Zhou, G. Comparative genome analysis of jujube witches'-broom phytoplasma, an obligate pathogen that causes jujube witches'-broom disease. *BMC Genomics* **2018**, 19, 689.
34. Xue, C.; Zhang, Y.; Li, H.; Liu, Z.; Gao, W.; Liu, M.; Wang, H.; Liu, P.; Zhao, J. The genome of *Candidatus phytoplasma* ziziphi provides insights into their biological characteristics. *BMC Plant Biology* **2023**, 23, 251.
35. Martini, M.; Lee, I.-M.; Bottner, K.D.; Zhao, Y.; Botti, S.; Bertaccini, A.; Harrison, N.A.; Carraro, L.; Marcone, C.; Khan, A.J.; Osler, R. Ribosomal protein gene-based phylogeny for finer differentiation and classification of phytoplasmas. *Int. J. Syst. Evol. Microbiol*. **2007**, 57, 2037-2051.
36. Krstić, O.; Cvrković, T.; Marinković, S.; Jakovljević, M.; Mitrović, M.; Toševski, I.; Jović, J. Genetic diversity of flavescence dorée phytoplasmas in vineyards of serbia: from the widespread occurrence of autochthonous map-M51 to the emergence of endemic map-FD2 (vectotype II) and new map-FD3 (vectotype III) epidemic genotypes. *Agronomy* **2022**, 12, 448.
37. Rigamonti, I.E.; Salvetti, M.; Girgenti, P.; Bianco, P.A.; Quaglino, F. Investigation on Flavescence dorée in north-western Italy identifies map-M54 (16SrV-D/map-FD2) as the only phytoplasma genotype in *Vitis vinifera* L. and reveals the presence of new putative reservoir plants. *Biology* **2023**, 12, 1216.
