# Supplementary material for: Occurrence, Impact, and Multilocus Sequence Analysis of Alder Yellows Phytoplasma Infecting Common Alder and Italian Alder in Southern Italy
Source: Microorganisms. 2024 Jun 4;12(6):1140. doi: 10.3390/microorganisms12061140 (PMC11205446; doi:10.3390/microorganisms12061140)
Supplement: Supplementary file 1 [file microorganisms-12-01140-s001.zip › Supplementary Figures-3a.pdf]

Supplementary Figures

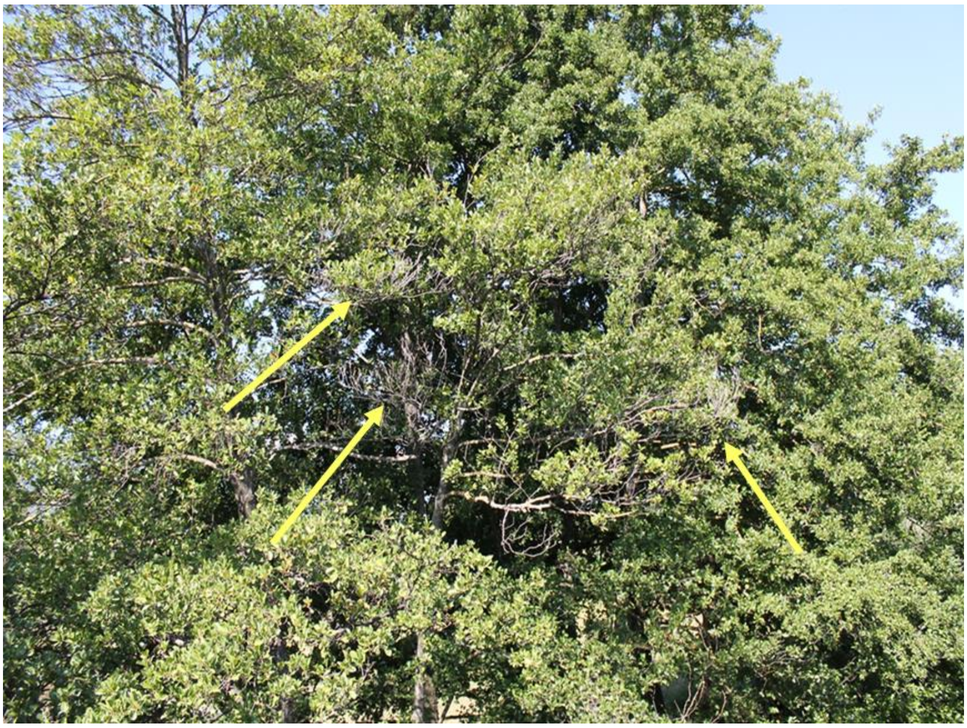

Figure S1. Premature shoots conferring a witches'-broom appearance on common alder trees infected by alder yellows (ALY) phytoplasma. These shoots do not lignify, are easily killed by the winter frost and are evident during the following year when they appear as dried twigs (arrows).

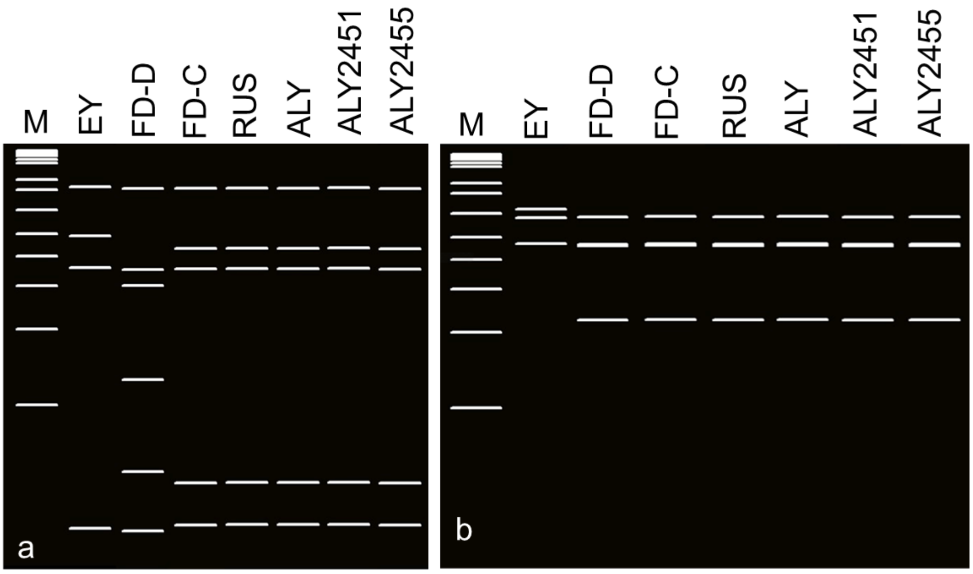

Figure S2. (**a**) *Taq*I and (**b**) *Bfa*I virtual restriction profiles of rDNA P1A/P7A fragments from alder yellows (ALY) phytoplasma strains and other 16SrV group phytoplasmas generated by AcaClone Software (http://www.acaclone.com). M, 1 Kb Plus DNA ladder; EY, elm yellows (strain EY1); FD-D, flavescence dorée (subgroup 16SrV-D, strain FD1487); FD-C, flavescence dorée (subgroup 16SrV-C, strain FD70); RUS, rubus stunt (strain RUS); ALY, alder yellows (strain ALY-SI); ALY2451 and ALY2455, newly recorded ALY phytoplasma strains in southern Italy (examples). The virtual gels were labelled and cropped with the software program Photoshop CS3 (www.adobe.com).

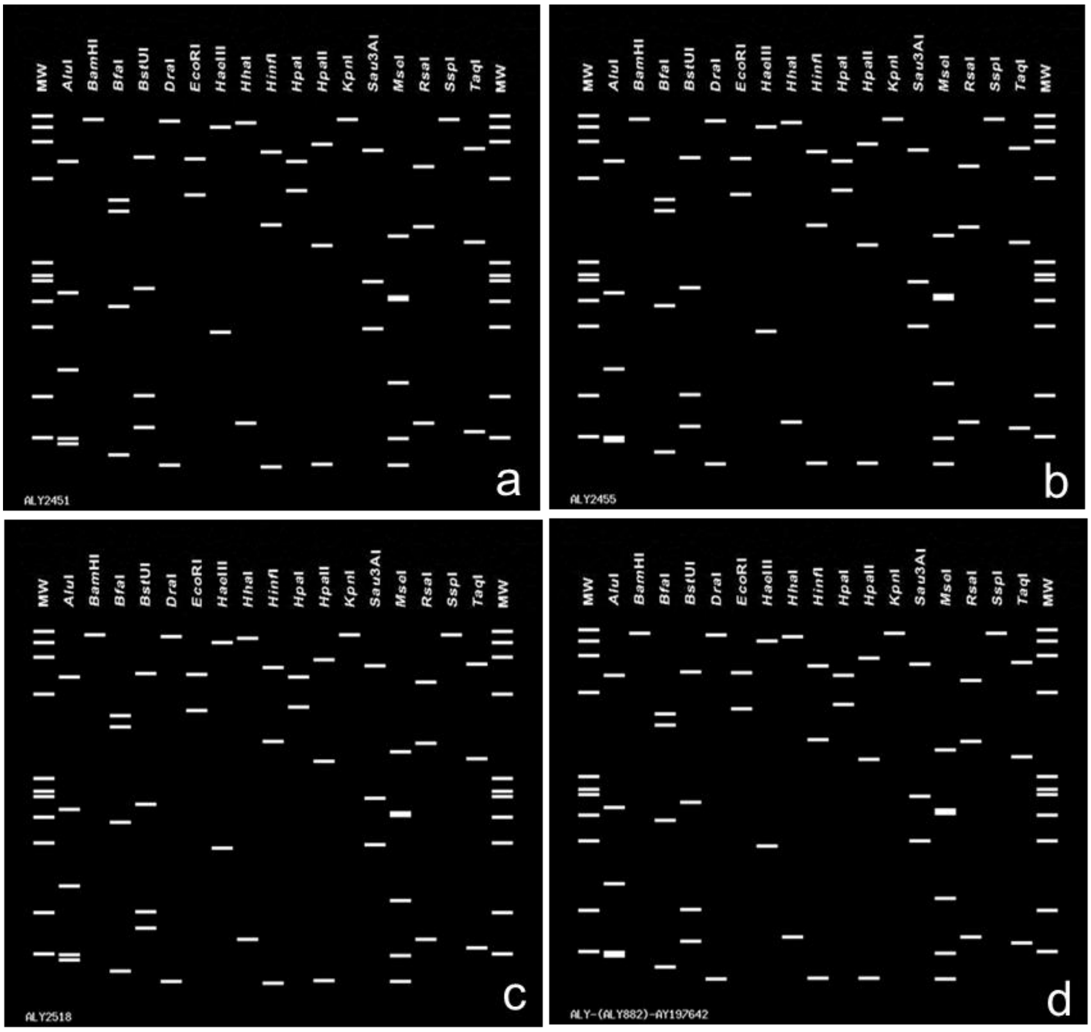

Figure S3. (**a**) through (**d**). Virtual RFLP analysis performed through the *i*PhyClassifier online tool at http://plantpathology.ba.ars.usda.gov/cgi-bin/resource/iphyclassifier.cgi [1] using 17 restriction enzymes of the 16S rDNA R16F2n/R2 fragments from ALY phytoplasma strains recorded in the present study (examples) and the phytoplasma reference strain ALY882. In (**c**), strain ALY2518 has a slightly different *Bst*UI restriction pattern. It is a variant of 16SrV-C subgroup. MW, *φ*X174DNA *Hae*III digest. The virtual gels were labelled and cropped with the software program Photoshop CS3 (www.adobe.com).

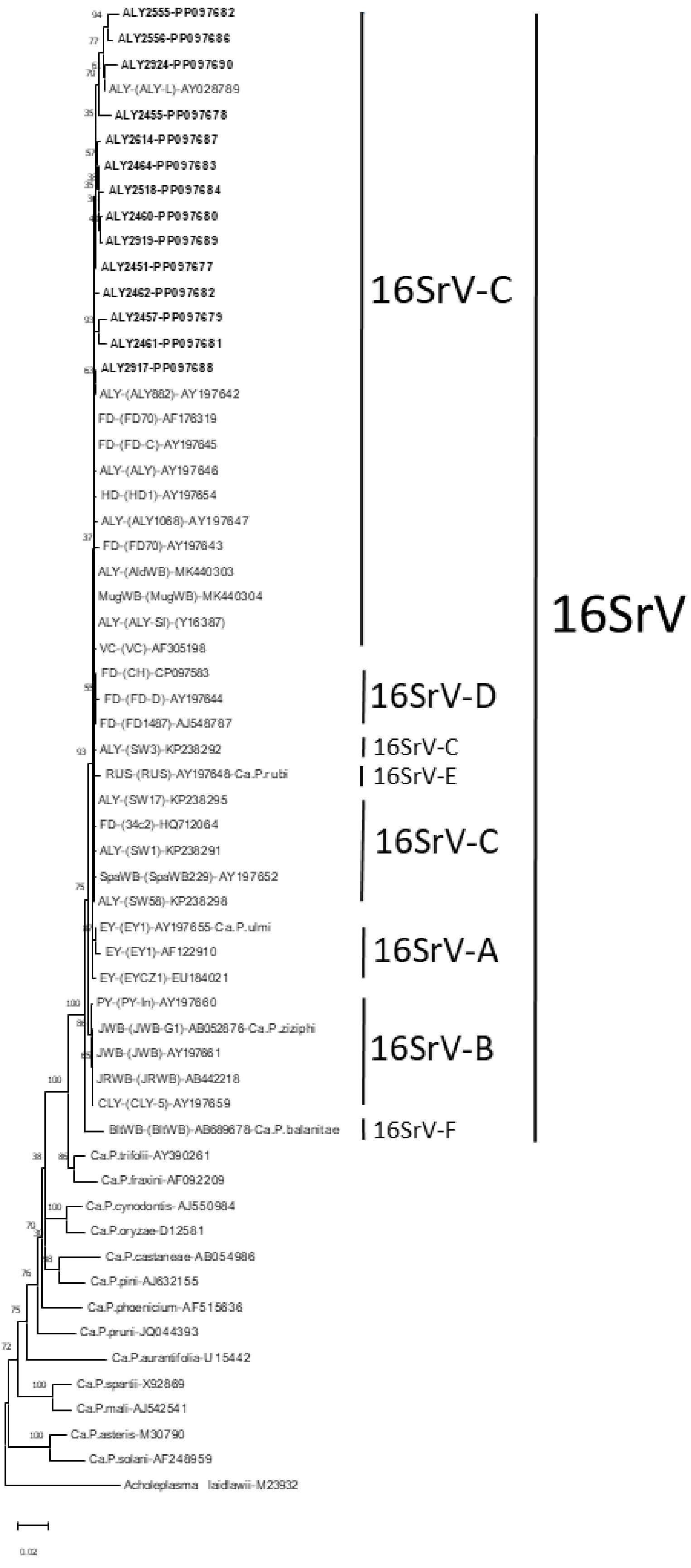
ALY2555-PP097682
ALY2556-PP097686
ALY2924-PP097690
ALY-(ALY-L)-AY028789
ALY2455-PP097678
ALY2614-PP097687
ALY2464-PP097683
ALY2518-PP097684
ALY2460-PP097680
ALY2919-PP097689
ALY2451-PP097677
ALY2462-PP097682
ALY2457-PP097679
ALY2461-PP097681
ALY2917-PP097688
ALY-(ALY882)-AY197642
FD-(FD70)-AF176319
FD-(FD-C)-AY197645
ALY-(ALY)-AY197646
HD-(HD1)-AY197654
ALY-(ALY1068)-AY197647
FD-(FD70)-AY197643
ALY-(AldWB)-MK440303
MugWB-(MugWB)-MK440304
ALY-(ALY-SI)-(Y16387)
VC-(VC)-AF305198
FD-(CH)-CP097583
FD-(FD-D)-AY197644
FD-(FD1487)-AJ548787
ALY-(SW3)-KP238292
RUS-(RUS)-AY197648-Ca.P.rubi
ALY-(SW17)-KP238295
FD-(34c2)-HQ712064
ALY-(SW1)-KP238291
SpaWB-(SpaWB229)-AY197652
ALY-(SW58)-KP238298
EY-(EY1)-AY197655-Ca.P.ulmi
EY-(EY1)-AF122910
EY-(EYCZ1)-EU184021
PY-(PY-In)-AY197660
JWB-(JWB-G1)-AB052876-Ca.P.ziziphi
JWB-(JWB)-AY197661
JRWB-(JRWB)-AB442218
CLY-(CLY-5)-AY197659
BltWB-(BltWB)-AB689678-Ca.P.balanitae
Ca.P.trifolii-AY390261
Ca.P.fraxini-AF092209
Ca.P.cynodontis-AJ550984
Ca.P.oryzae-D12581
Ca.P.castaneae-AB054986
Ca.P.pini-AJ632155
Ca.P.phoenicium-AF515636
Ca.P.pruni-JQ044393
Ca.P.aurantifolia-U15442
Ca.P.spartii-X92869
Ca.P.mali-AJ542541
Ca.P.asteris-M30790
Ca.P.solani-AF248959
Acholeplasma laidlawii-M23932
16SrV-C
16SrV-D
16SrV-C
16SrV-E
16SrV-C
16SrV-A
16SrV-B
16SrV-F
16SrV
0.02

Figure S4. Phylogenetic tree constructed using the neighbor-joining method software MEGA, version XI (www.megasoftware.net) [2] with 16S rDNA sequences from newly detected ALY phytoplasma strains in southern Italy (in bold type), ALY strains previously detected in alder, other 16SrV-C subgroup members, 16SrV-A, -B, -D, -E and -F subgroup phytoplasmas, and a number of formally described '*Candidatus* Phytoplasma' species. *Acholeplasma laidlawii* was used as the outgroup. Bar represents a phylogenetic distance of 0.02 nucleotide substitutions per site. GenBank accession number is given for each phytoplasma. Bootstrap values are shown on branches of the phylogenetic tree.

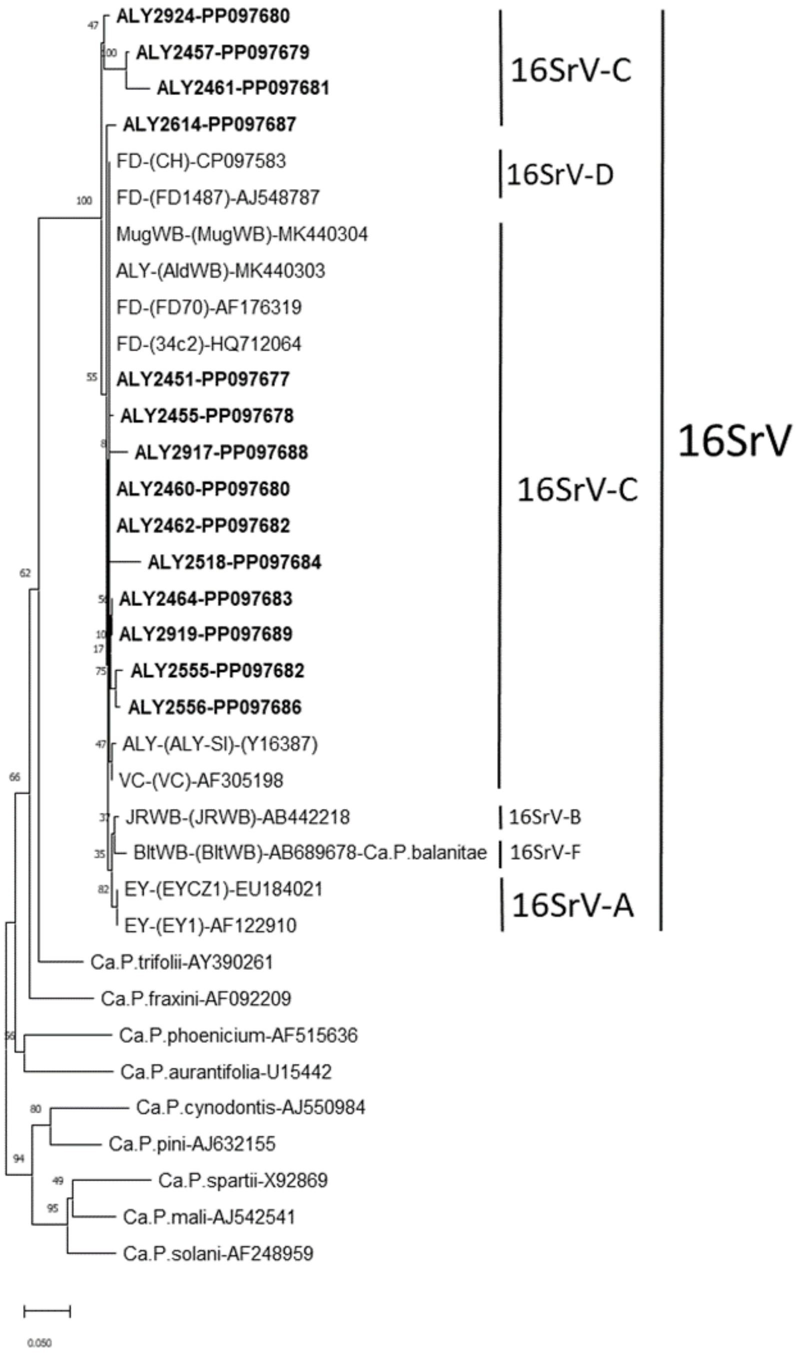

Figure S5. Phylogenetic tree constructed using the neighbor-joining method software MEGA, version XI (www.megasoftware.net) [2] with 16S/23S rDNA spacer region sequences from newly detected ALY phytoplasma strains in southern Italy (in bold type), ALY strains previously detected in alder, other 16SrV-C subgroup members, 16SrV-A, -B, -D, and -F subgroup phytoplasmas, and a number of formally described '*Candidatus* Phytoplasma' species. '*Ca.* Phytoplasma solani' was used as the outgroup. Bar represents a phylogenetic distance of 0.05 nucleotide substitutions per site. GenBank accession number is given for each phytoplasma. Bootstrap values are shown on branches of the phylogenetic tree.

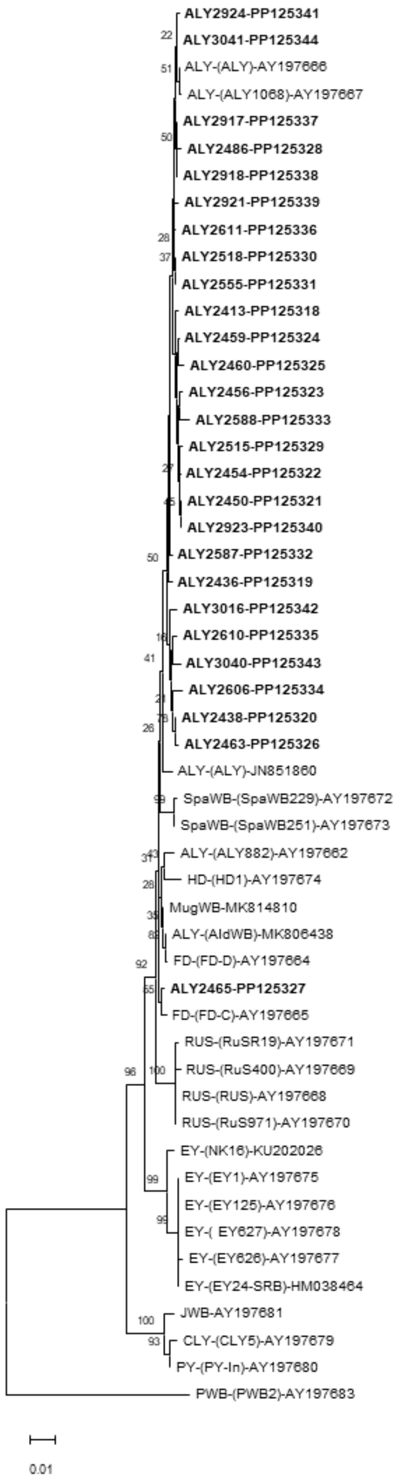
ALY2924-PP125341
ALY3041-PP125344
ALY-(ALY)-AY197666
ALY-(ALY1068)-AY197667
ALY2917-PP125337
ALY2486-PP125328
ALY2918-PP125338
ALY2921-PP125339
ALY2611-PP125336
ALY2518-PP125330
ALY2555-PP125331
ALY2413-PP125318
ALY2459-PP125324
ALY2460-PP125325
ALY2456-PP125323
ALY2588-PP125333
ALY2515-PP125329
ALY2454-PP125322
ALY2450-PP125321
ALY2923-PP125340
ALY2587-PP125332
ALY2436-PP125319
ALY3016-PP125342
ALY2610-PP125335
ALY3040-PP125343
ALY2606-PP125334
ALY2438-PP125320
ALY2463-PP125326
ALY-(ALY)-JN851860
SpaWB-(SpaWB229)-AY197672
SpaWB-(SpaWB251)-AY197673
ALY-(ALY882)-AY197662
HD-(HD1)-AY197674
MugWB-MK814810
ALY-(AldWB)-MK806438
FD-(FD-D)-AY197664
ALY2465-PP125327
FD-(FD-C)-AY197665
RUS-(RuSR19)-AY197671
RUS-(RuS400)-AY197669
RUS-(RUS)-AY197668
RUS-(RuS971)-AY197670
EY-(NK16)-KU202026
EY-(EY1)-AY197675
EY-(EY125)-AY197676
EY-(EY627)-AY197678
EY-(EY626)-AY197677
EY-(EY24-SRB)-HM038464
JWB-AY197681
CLY-(CLY5)-AY197679
PY-(PY-In)-AY197680
PWB-(PWB2)-AY197683
0.01

Figure S6. Phylogenetic tree constructed using the neighbor-joining method software MEGA, version XI (www.megasoftware.net) [2] with rp genes *rpsV* (*rpl22*) and *rpsC* (*rps3*) sequences from newly detected ALY phytoplasma strains in southern Italy (in bold type), ALY strains previously detected in alder, and flavescence dorée (FD), spartium witches'-broom (SpaWB), rubus stunt (RUS), elm yellows (EY), hemp dogbane yellows (HD), mugwort witches'-broom (MugWB), jujube witches'-broom (JWB), cherry lethal yellows (CLY) and peach yellows (PY) strains. Potato witches'-broom (PWB) phytoplasma strain PWB2 was used as the outgroup. Bar represents a phylogenetic distance of 0.01 nucleotide substitutions per site. GenBank accession number is given for each phytoplasma. Bootstrap values are shown on branches of the phylogenetic tree.

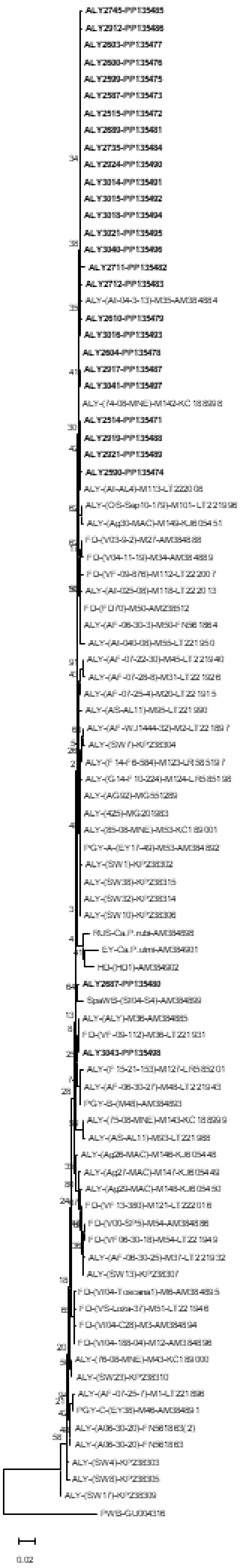

ALY2745-PP135485
ALY2012-PP135486
ALY2603-PP135477
ALY2600-PP135476
ALY2599-PP135475
ALY2587-PP135473
ALY2515-PP135472
ALY2689-PP135481
ALY2735-PP135484
ALY2024-PP135490
ALY3014-PP135491
ALY3015-PP135492
ALY3018-PP135494
ALY3021-PP135495
ALY3040-PP135496
ALY2711-PP135482
ALY2712-PP135483
ALY-(AI-04-3-13)-M35-AM384884
ALY2610-PP135479
ALY3016-PP135493
ALY2604-PP135478
ALY2017-PP135487
ALY3041-PP135497
ALY-(74-08-MNE)-M142-KC188998
ALY2514-PP135471
ALY2019-PP135488
ALY2021-PP135489
ALY2590-PP135474
ALY-(AI-AL4)-M113-LT222008
ALY-(OS-Sep10-179)-M101-LT221998
ALY-(Ag30-MAC)-M149-KJ605451
FD-(V03-9-2)-M27-AM384888
FD-(V04-11-19)-M34-AM384889
FD-(VF-09-878)-M112-LT222007
ALY-(AI-025-08)-M118-LT222013
FD-(FD70)-M50-AM238512
ALY-(AF-08-30-3)-M50-FN561884
ALY-(AI-040-08)-M55-LT221950
ALY-(AF-07-22-30)-M45-LT221940
ALY-(AF-07-28-8)-M31-LT221926
ALY-(AF-07-25-4)-M20-LT221915
ALY-(AS-AL11)-M95-LT221990
ALY-(AF-WJ1444-32)-M2-LT221897
ALY-(SW7)-KP238304
ALY-(F14-F8-584)-M123-LR585197
ALY-(G14-F10-224)-M124-LR585198
ALY-(AG92)-MG551289
ALY-(425)-MG201983
ALY-(85-08-MNE)-M53-KC189001
PGY-A-(EY17-40)-M53-AM384892
ALY-(SW1)-KP238302
ALY-(SW38)-KP238315
ALY-(SW32)-KP238314
ALY-(SW10)-KP238306
RUS-Ca.P.rubi-AM384898
EY-Ca.P.ulmi-AM384901
HD-(HD1)-AM384902
ALY2687-PP135480
SpaWB-(SI04-S4)-AM384899
ALY-(ALY)-M36-AM384885
FD-(VF-09-112)-M36-LT221931
ALY3043-PP135498
ALY-(F15-21-153)-M127-LR585201
ALY-(AF-08-30-27)-M48-LT221943
PGY-B-(M48)-AM384893
ALY-(75-08-MNE)-M143-KC188999
ALY-(AS-AL11)-M93-LT221988
ALY-(Ag28-MAC)-M146-KJ605448
ALY-(Ag27-MAC)-M147-KJ605449
ALY-(Ag29-MAC)-M148-KJ605450
FD-(VF13-380)-M121-LT222016
FD-(V00-SP5)-M54-AM384886
FD-(VF08-30-18)-M54-LT221949
ALY-(AF-08-30-25)-M37-LT221932
ALY-(SW13)-KP238307
FD-(VI04-Toscana1)-M8-AM384895
FD-(VS-Loza-37)-M51-LT221946
FD-(VI04-C28)-M3-AM384894
FD-(VI04-188-04)-M12-AM384896
ALY-(76-08-MNE)-M43-KC189000
ALY-(SW23)-KP238310
ALY-(AF-07-25-7)-M1-LT221896
PGY-C-(EY38)-M46-AM384891
ALY-(A08-30-20)-FN561863(2)
ALY-(A08-30-20)-FN561863
ALY-(SW4)-KP238303
ALY-(SW8)-KP238305
ALY-(SW17)-KP238309
PWB-GU004316
0.02

Figure S7. Phylogenetic tree constructed using the neighbor-joining method software MEGA, version XI (www.megasoftware.net) [2] with *map* gene sequences from newly detected ALY phytoplasma strains in southern Italy (in bold type), ALY strains previously detected in alder, and flavescence dorée (FD), Palatinate grapevine yellows (PGY), spartium witches'-broom (SpaWB), rubus stunt (RUS), elm yellows (EY) and hemp dogbane yellows (HD) strains. Potato witches'-broom (PWB) phytoplasma strain PWB was used as the outgroup. Bar represents a phylogenetic distance of 0.02 nucleotide substitutions per site. GenBank accession number is given for each phytoplasma. Bootstrap values are shown on branches of the phylogenetic tree.

References

1. Zhao, Y.; Wei, W.; Lee, I.-M.; Shao, J.; Suo, X.; Davis, R.E. Construction of an interactive online phytoplasma classification tool, *i*PhyClassifier, and its application in analysis of the peach X-disease phytoplasma group (16SrIII). *Int. J. Syst. Evol. Microbiol.* **2009**, 59, 2582-2593.
2. Tamura, K.; Stecher, G.; Kumar, S. MEGA 11: Molecular evolutionary genetics analysis version 11. *Mol. Biol. Evol.* **2021**, 38 (7), 3022-3027.
